# New Challenges in Targeting Signaling Pathways in Acute Lymphoblastic Leukemia by NGS Approaches: An Update

**DOI:** 10.3390/cancers10040110

**Published:** 2018-04-07

**Authors:** Adrián Montaño, Maribel Forero-Castro, Darnel Marchena-Mendoza, Rocío Benito, Jesús María Hernández-Rivas

**Affiliations:** 1 IBSAL, IBMCC, Universidad de Salamanca-CSIC, Cancer Research Center, 37007 Salamanca, Spain; adrianmo18@gmail.com (A.M.); darnelmarchena@hotmail.com (D.M.-M.); beniroc@usal.es (R.B.); 2 Escuela de Ciencias Biológicas, Grupo de investigación en Ciencias Biomédicas (GICBUPTC), Universidad Pedagógica y Tecnológica de Colombia, Tunja 150001, Colombia; maribel.forero@uptc.edu.co; 3 Universidad de Salamanca-CSIC, Hospital Universitario de Salamanca, 37007 Salamanca, Spain

**Keywords:** NGS, mutations, fusion genes, pathways

## Abstract

The identification and study of genetic alterations involved in various signaling pathways associated with the pathogenesis of acute lymphoblastic leukemia (ALL) and the application of recent next-generation sequencing (NGS) in the identification of these lesions not only broaden our understanding of the involvement of various genetic alterations in the pathogenesis of the disease but also identify new therapeutic targets for future clinical trials. The present review describes the main deletions, amplifications, sequence mutations, epigenetic lesions, and new structural DNA rearrangements detected by NGS in B-ALL and T-ALL and their clinical importance for therapeutic procedures. We reviewed the molecular basis of pathways including transcriptional regulation, lymphoid differentiation and development, TP53 and the cell cycle, RAS signaling, JAK/STAT, NOTCH, PI3K/AKT/mTOR, Wnt/β-catenin signaling, chromatin structure modifiers, and epigenetic regulators. The implementation of NGS strategies has enabled important mutated genes in each pathway, their associations with the genetic subtypes of ALL, and their outcomes, which will be described further. We also discuss classic and new cryptic DNA rearrangements in ALL identified by mRNA-seq strategies. Novel cooperative abnormalities in ALL could be key prognostic and/or predictive biomarkers for selecting the best frontline treatment and for developing therapies after the first relapse or refractory disease.

## 1. Introduction

Acute lymphoblastic leukemia (ALL) is a malignant disorder originating from hematopoietic B- or T-cell precursors and is characterized by marked heterogeneity at the molecular and clinical levels. B-ALL and T-ALL comprise multiple subtypes defined by their primary chromosomal abnormality (mainly chromosomal translocations that give rise to chimeric fusion genes or broad aneuploidy) and defined by the cooperating secondary aberrations (deletions, amplifications, sequence mutations, and epigenetic lesions), which jointly contribute to leukemogenesis [1,2]. 

Although a genetic event is known to occur in the majority of cases and may be associated with outcome prediction, around 25% to 30% of pediatric and 50% of adult ALL patients have no defined genetic hallmarks of biological or clinical significance [1]. Given the increasing number of these alterations, cytogenetics and FISH, which are methods commonly used in molecular biology, as well as small sequencing panels that focus on a limited number of genes may not be sufficient to identify new cryptic lesions especially those in lymphoid malignancies [3]. 

The introduction and rapid spread of DNA copy-number analysis and genome-wide DNA/RNA sequencing analysis have shown that ALL subgroups are characterized by numerous cooperating oncogenic lesions that affect genes with an established role in the proliferation and establishment of the leukemic clone. Recently, next-generation sequencing (NGS) technologies have identified many novel lesions in ALL and helped not only improve our understanding of its pathogenesis, but also helped to discover key biomarkers of diagnostic and prognostic importance [4]. The concept of NGS involves DNA, RNA, or miRNA sequencing through various approaches such as targeted sequencing, whole-exome sequencing (WES), transcriptome sequencing (messenger mRNA [mRNA-seq]), whole-genome sequencing (WGS), and epigenomics [5]. 

These technologies have enabled new genetic alterations to be characterized that include somatic structural DNA rearrangements, deletions of genes involved in differentiation and cell-cycle control, fusion genes that target B- or T-cell differentiation, and mutations (e.g., recurrent substitutions, indels) of genes. These mutations play crucial roles in key signaling pathways associated with the pathogenesis of ALL [1,5,6]. Similarly, many other genes become activated or inactivated due to the presence of specific somatic mutations (point or indel) or complex structural rearrangements or are affected by copy-number changes (deletions and/or amplifications) [7]. 

For this reason, next-generation sequencing is an important tool for detecting key genetic alterations, which directly conditions the prognosis, pathogenesis, and the evolution of patients. However, the clinical NGS applications come with great limitations. One of them is that NGS requires sophisticated bioinformatics systems, fast data processing, and large data storage capabilities, which can be costly. However, data interpretation remains a significant challenge due to the large number of variants detected by the huge molecular heterogeneity of the diseases.

In this review, we describe the alterations at the genetic level in the various signaling pathways associated with the pathogenesis of acute lymphoblastic leukemia (ALL) and the application of recent next-generation sequencing (NGS) not only for identifying and studying these lesions but also for identifying new therapeutic targets for future clinical trials.

## 2. Genomic Heterogeneity in Molecular Subtypes of ALL

The WHO classification divides ALL in relation to the presence of primary genetic abnormalities and it is well known that the incidence, clinical, and biological characteristics of these alterations vary with age. Additionally, many of the characteristics are strong, independent predictive factors of outcome [8,9]. As a consequence of implementation of new genomic technologies, new subtypes of ALL have been recognized in the 2016 revision of the WHO classification of myeloid neoplasms and acute leukemia [10]. This latest revision helps improve our understanding of the biology and molecular pathogenesis of ALL as well as the heterogeneity of its genetic subtypes [8,10]. Particularly, two new entities known as intrachromosomal amplification of chromosome 21 (iAMP21) and *BCR*-*ABL*-like B lymphoblastic leukemia/lymphoma have been recognized in the ‘B lymphoblastic leukemia/lymphoma with recurrent genetic abnormalities’ category [10]. A novel subgroup of T-ALL called immature early T-cell precursor leukemia (ETP-ALL) is the only subtype of T-ALL recognized as being a new entity in the 2016 WHO revision [3,10,11]. Supplemental file 1 shows the frequency and prognosis value of cytogenetic and molecular genetic abnormalities identified in ALL.

Based on the gene-expression profile analysis, in 2009 Den Boer et al. [12] and Mullighan et al. [13] independently identified a new high-risk subgroup of B-ALL called *BCR*-*ABL*-like or Ph-like. This group is characterized by a biological profile similar to the Philadelphia chromosome/*BCR-ABL* positive patients (Ph+) but without *BCR-ABL* fusion. It shares the same high risk of relapse and worse clinical outcome and responsiveness to tyrosine kinase inhibitors (TKIs) [8]. This subtype accounts for 15% of B-ALL, which comprises the 10% of cases of childhood B-ALL and 20% of the cases of adult B-ALL with a peak prevalence of 28% in young adults (aged 21 to 39 years) [1,14]. Recently with NGS-based methods, a spectrum of genetic abnormalities have been detected in BCR-ABL-like ALL that extends our understanding of this leukemic subgroup, but because these are still no determinants in the diagnosis, other techniques such as FISH remain necessary. These cooperating lesions comprise the somatic mutations and gene fusions that are the most frequent lesions associated with pathogenesis of Ph-like ALL [15]. Common genomic lesions of *BCR-ABL*-like ALL include the B-lymphoid transcription factor genes (particularly *IKZF1* deletions), somatic mutations in JAK-STAT and RAS signaling (*NRAS*, *KRAS*, *PTPN11*, *NF1*), and, less commonly, kinase alterations (*FLT3*, *NTRK3*, *BLNK*, *TYK2*, *PTK2B*). Structural rearrangements have also been identified in this ALL subgroup, which affects the *CRLF2* gene, ABL-class tyrosine kinase genes, and *JAK2* and *EPOR* genes [1].

Intrachromosomal amplification of chromosome 21 (iAMP21) is a distinct subgroup of childhood ALL that is present in 2% of older children and has also been associated with a poorer outcome that improves with intensive treatment [1,10]. This abnormality has been confirmed to be a primary genetic event in B-ALL [16] and has been identified when three or more additional copies of *RUNX1* (*AML1*) are observed on a single abnormal chromosome 21 (including a total of five or more *RUNX1* copies per cell) [10,17]. iAMP is a distinct marker caused by the breakage-fusion-bridge cycle and chromothripsis, which involves tens to hundreds of genomic rearrangements with multiple regions of gain, amplification, inversion, and deletion [10,18,19]. 

Finally, ETP-ALL expressing ETP/stem cell genes were recently identified by whole-genome expression profiling. This new T-ALL subgroup is characterized by a high mutation load and worse survival rates than those of other T-ALL subgroups [3,10,11]. Multiple recurrent genomic lesions have recently been identified in this heterogeneous subgroup in which notable examples include the aberrant expression of the transcription factor *LYL1* and the t(2;14)(q22;q32) translocation that affects the *BCL11B* (14q32) and *ZEB2* (2q22) genes. This results in the sustained overexpression of *ZEB2*, which enhances the leukemic stem cell properties [11,20].

## 3. The Mutational Landscape in Signaling Pathways Involved in ALL

Somatic mutations drive oncogenic processes that promote the arrest of differentiation or can contribute to leukemogenesis by altering a wide range of cellular processes (cell cycle, epigenetic gene regulation, and apoptosis), which ultimately leads to the constitutive activation of crucial intracellular pathways associated with pathogenesis of ALL [21]. In recent years, various NGS studies of childhood and adult B-ALL and T-ALL have revealed a spectrum of somatic mutations in several genes involved in multiple canonical and non-canonical signaling pathways of ALL, such us transcriptional regulation, lymphoid differentiation and development, *TP53* and the cell cycle, *RAS* signaling, *JAK*/*STAT*, *NOTCH*, *PI3K/AKT/mTOR*, Wnt/β-catenin signaling, chromatin structure modifiers, and epigenetic regulators.

**Figure 1:**
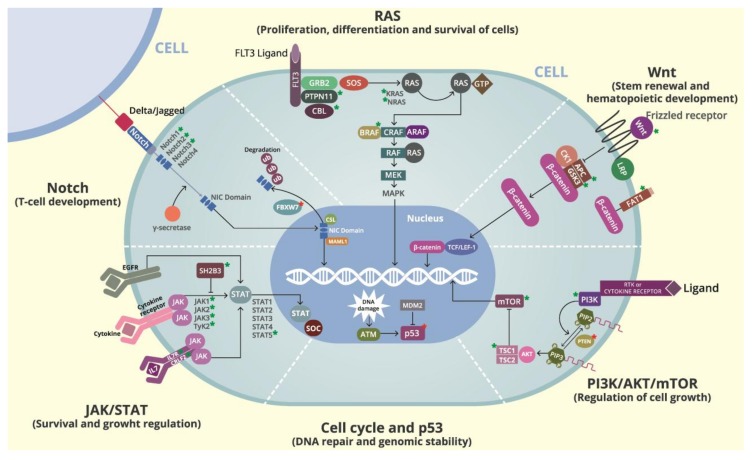
Cellular pathways affected by genes significantly mutated in ALL. Asterisks show the genes that are mutated in each cellular program and the biological effect of these mutations on the pathway (Green: activation mutation; Red: inactivating mutation).

**Figure 2:**
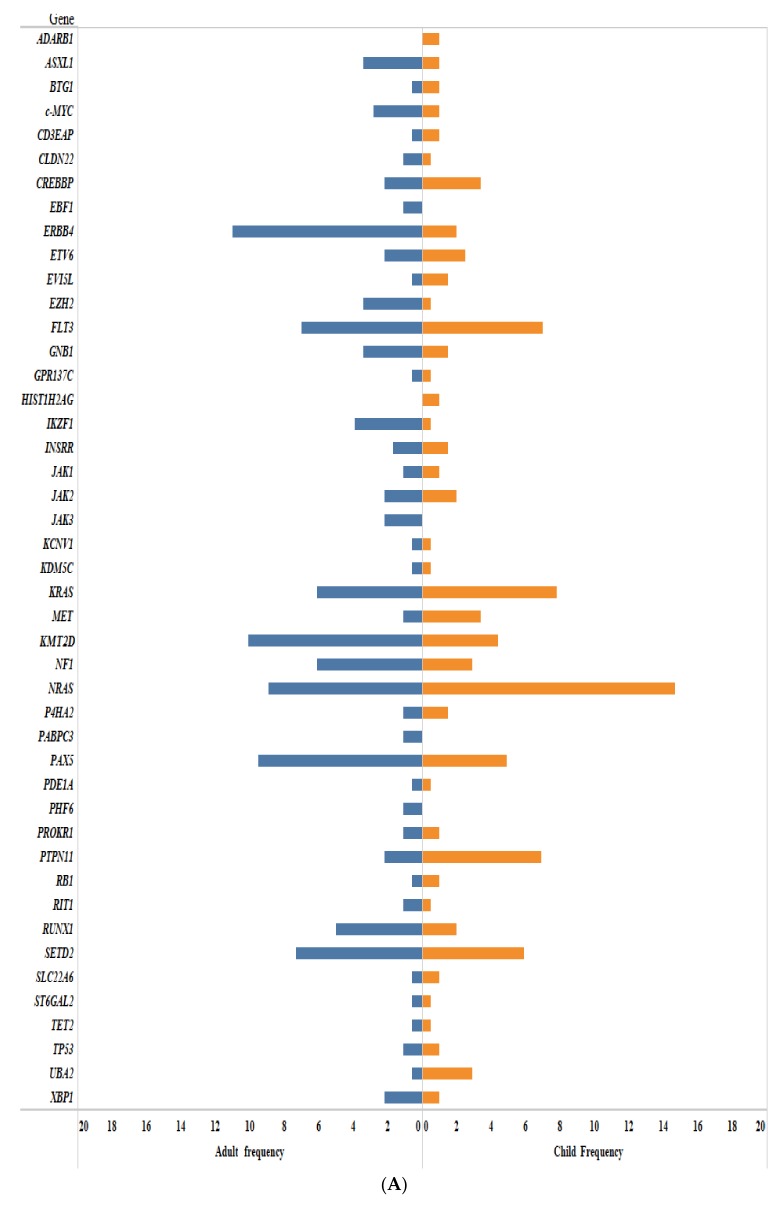

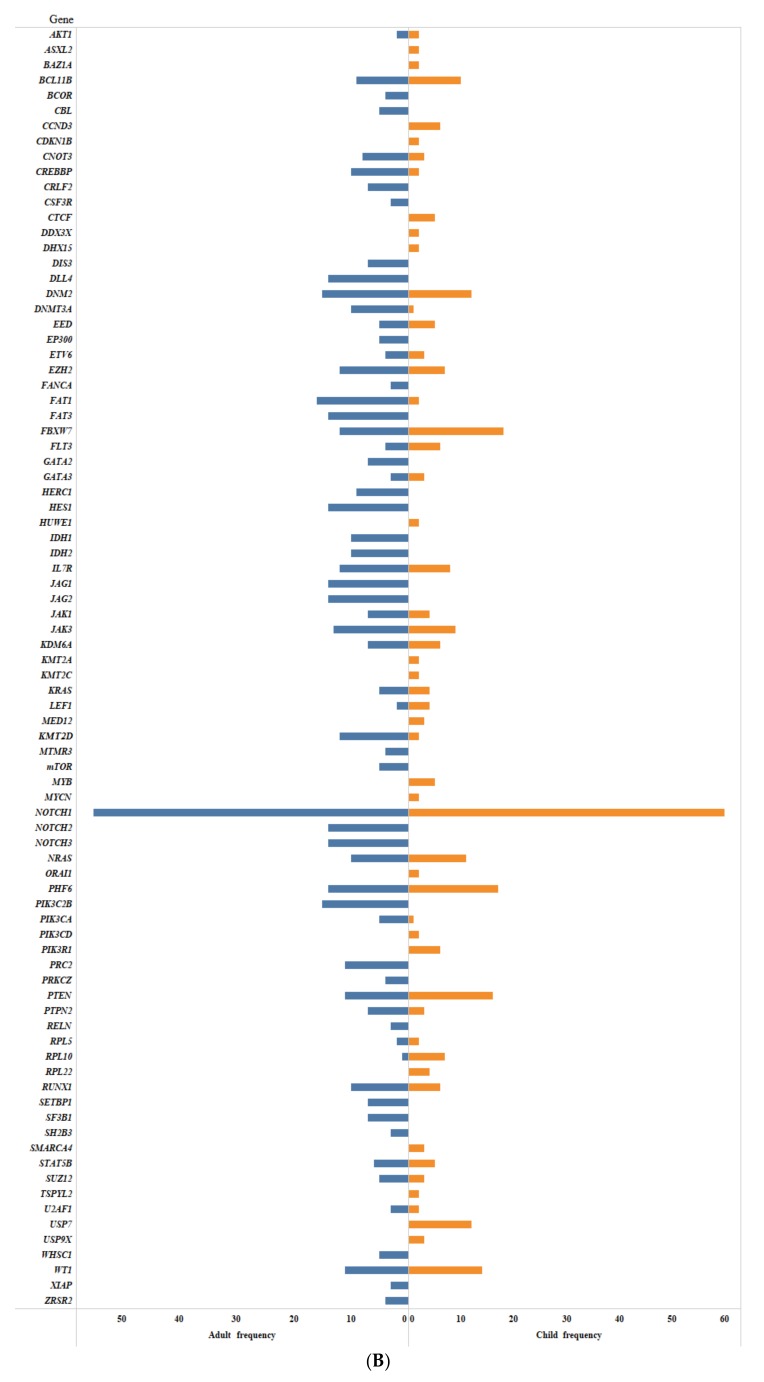
(**A**) Frequency of somatic mutations identified by NGS methods in B-ALL. The figure shows the frequency of somatic mutations in childhood and adult B-ALL. (**B**) Frequency of somatic mutations identified by NGS methods in T-ALL. The figure shows the frequency of somatic mutations in childhood and adult T-ALL.

In particular, somatic mutations in several genes have been identified by NGS in B-ALL. These include genes associated with B-cell differentiation and development (18%; e.g., *PAX5*, *IKZF1*, *EBF1*), RAS signaling (48%; e.g., *NRAS*, *KRAS*, *PTPN11*, *FLT3*, *NF1*), JAK/STAT signaling (11%; e.g., *JAK1*, *JAK2*, *IL7R*, *CRLF2*), cell cycle regulation and tumor suppression (6%; e.g., *TP53*, *RB1*, *CDKN2A/B*), and non-canonical pathways or other/unknown genes (17%; e.g., *CREBBP*, *NT5C2*, *TBL1XR1* [22,23]. At the same time, in T-ALL, somatic mutations in the *NOTCH* signaling pathway (60%; e.g., *NOTCH1*, *NOTCH2*, *NOTCH3*, *FBXW7*, *HES1*, *JAG1*, *JAG2*), transcription factors (9–17%; e.g., *LMO2*, *TAL1(SCL)*, *TLX3*, *WT1*, *BCL11B*, *LEF1*, *ETV6*), RAS signaling (15%; e.g., *NRAS*, *KRAS*, *FLT3*, *CBL*), JAK/STAT signaling (20–30%; e.g., *JAK1*, *JAK2*, *JAK3*, *IL7R*, *STAT5B*) [24,25], PI3K/AKT/mTOR signaling (85%; e.g., *PIK3CA*, *AKT1*, *mTOR*, *PTEN*), Wnt/β-catenin signaling pathway (13%; e.g., *FAT1*, *FAT3*, *LEF1*) [24], chromatin structure modifiers and epigenetic regulators (35%; e.g., *PHF6*, *EZH2*, *DNMT3A*) [24], ribosomal processes (*RPL5*, *RPL10*, *RPL22* [25,26]), and recently discovered novel recurrent mutations in the DNA repair complex (*HER1/EGFR*), splicing factor (*ZRSR2*) [24], and various other functions (e.g., *WT1*, *CNOT3* [26], *NT5C2*, *MEF2C*) [11,24,25,27,28]. Figure 1 illustrates the pathways and significantly mutated genes in ALL and Figure 2 shows the frequency of somatic mutations identified in ALL by using NGS methods. Supplemental file 2 details the references of Figure 2.

### 3.1. Transcriptional Regulation and Lymphoid Differentiation and Development 

Gene transcription plays a critical role in controlling normal hematopoietic differentiation. Transcription factors strongly influence cellular lineage during hematopoiesis and their genetic alterations can alter the normal mechanisms that control lymphoid differentiation and development. Lymphoid cells are derived from pluripotent hematopoietic stem cells present in the bone marrow through stepwise maturation. This process is tightly controlled by the hierarchical activation of transcription factors and selection through functional signal transduction. The emergence of secondary genetic alterations such as deletions, amplifications, mutations, and structural rearrangements in key transcription factors disrupts lymphoid development and results in the arrest of maturation of B-lineages and T-lineages [24,29]. 

Important transcriptional factors involved in lymphoid development and differentiation (*EBF1*, *ETV6*, *PAX5*, *RUNX1*, and *TCF3*) participate in structural rearrangements. Many of these transcriptional factors are identified by RNA-sequencing methods, which will be described later. 

NGS studies have found somatic mutations in different transcription factors involved in the pathogenesis of T-ALL such as inactivating mutations in *BCL11B*, *ETV6*, *GATA3*, *LEF1*, *RUNX1,* and *WT1* genes [11,25]. Somatic mutations in *BTG1*, *c-MYC*, *ERG*, *ETV6*, *IKZF1*, *IKZF2*, *IKZF3*, *LEF1*, *PAX5,* and *TBL1XR1* have been detected in B-ALL [29]. Sequencing the *IKZF1* gene has revealed a low frequency of somatic point mutations in B-ALL [13]. However, inherited genetic variants and rare deleterious mutations in the *IKZF1* gene play a role in the risk of developing B-ALL. *IKZF1* encodes the transcription factor IKAROS, which is indispensable for the induction of B-lineage differentiation in hematopoietic stem cells. As shown in murine models, their mutations have been recognized as being some of the most detrimental driver mutations in ALL by accelerating the onset of B-ALL in in vivo assays [1,4,29]. Deletion and mutation of other genes essential to B-cell development including *EBF1*, *RAG1*, *RAG2* and *LEF1* are also frequently detected in B-ALL [30].

Acquired somatic lesions in transcription factors correspond to non-synonymous single-nucleotide substitutions as well as frameshift and nonsense changes [11]. However, some genes such as *BCL11B*, *ETV6*, *ERG*, *GATA3*, *LEF1*, *LMO2*, *RUNX1*, *TAL1(SCL)*, *TLX1*, and *WT1* harbor some deletions in ALL cases that are difficult to detect by using NGS methods.

Focal deletions and sequence mutations in the *IKZF1* gene have been found in 15% of pediatric B-ALL and more than 80% of *BCR-ABL* cases. Recurrent mutations of *PAX5* occur in about one-third of B-ALL cases and in up to 50% of *BCR-ABL* positive cases. *EBF1* mutations occur in about 14% of *BCR-ABL* positive cases [31]. More than 80% of *BCR-ABL*-like patients have one or more alterations in genes involved in B-cell development including *IKAROS (IKZF1)*, *E2A (TCF3)*, *EBF1*, *PAX5*, and *VPREB1* [12].

*IKZF2* mutations are hallmarks of low hypodiploid B-ALL. On the other hand, most cases of near-haploid ALL have a high frequency of *IKZF3* alterations [5,32]. *ETV6* mutations are found in rare B-ALL cases [30]. Deletions in *IKZF1*, *CDKN2A*, *PAX5*, *ETV6*, *RB1*, and *TCF3* genes or unbalanced translocations have been observed in high hyperdiploid B-ALL [33]. Loss-of-function mutations in transcription factors acting as hematopoietic regulators (*GATA3*, *IKZF1*, *RUNX1*, *ETV6*) have been described in ETP-ALL [5,11,27,34].

Clinical studies have demonstrated that *LEF1* mutations are associated with a favorable outcome in ALL [35]. By contrast, *IKZF1* alterations (mutation/deletion) confer resistance to therapy and promote disease relapse [4]. Similarly, *RUNX1* mutations have been associated with poor outcomes in ALL. These mutations are generally distributed across several exons but are mainly clustered in the RUNT domain (amino acids 50–177; e.g., Cys72Tyr and Arg174X) and less frequently in the TAD domain (amino acids 291–371; e.g., Pro332_Val333insTrpGly and Ile337ValfsX231) [36,37]. In particular, the high incidence of *RUNX1* mutations in early T-ALL makes the gene a novel biomarker for T-ALL because the mutations have revealed its important role in early hematopoietic development [36,37].

On the other hand, it should be noted that the B-cell receptor signaling pathway (pre-BCR signaling) is a crucial pathway early on in B-cell development. The *BTK* gene, which is essential for B-cell development, participates in this pathway. This gene is deregulated in pre-BCR ALL patients. Pre-B signaling leads to leukemic cell proliferation except in some cells that have a secondary alteration, which occurs in ALL *BCR-ABL*-positive cases. Around 15% of ALL patients are dependent on pre-BCR signaling and have been shown to be sensitive to BCR inhibitors [38,39,40]. 

### 3.2. RAS Signaling Pathway

The RAS signaling pathway is important for the proliferation, differentiation, and survival of the cell. Alterations in its function promote tumor transformation. Three *RAS* genes participate in this pathway including *H-RAS*, *K-RAS*, and *N-RAS*. RAS proteins are activated by guanine-nucleotide exchange factors (GEFs), inactivated by hydrolysis of GTP due to its intrinsic GTPase activity, and accelerated by the action of GTPase-activating protein (GAP). RAS activation is initiated by tyrosine kinase receptors like the epithelial growth factor receptor (EGFR), the platelet-derived growth factor receptor, and the insulin receptor. Once activated, RAS can orchestrate its effects through three major downstream pathways including the RAF family protein (C-Raf, B-Raf, A-Raf), which activates the mitogen-activated protein kinase (MAPK) to promote cell proliferation, differentiation, survival, inflammation, and the inhibition of apoptosis. Lastly, the RAS-like guanine nucleotide-dissociation stimulator (RalGDS) cascade participates in a wide variety of cellular processes [41,42] (see Figure 1). 

NGS studies have identified oncogenic gain-of-function mutations in the *KRAS*, *NRAS*, *PTPN11*, *BRAF*, *FLT3*, *CBL*, and *CBLB* genes as well as loss of function mutations in the *NF1* gene, which is a negative regulator of the RAS pathway in patients with ALL [24,42,43]. The recurrent mutations in the RAS pathway involve the *KRAS* and *NRAS* genes [41]. Downstream or upstream mutations in elements of the RAS pathway constitutively activate RAS signaling by cooperating in B-lineage and T-lineage leukemogenesis [42].

Mutations in RAS genes involve mainly single nucleotide substitutions that generate missense and frameshift variations [41,43,44]. Activating mutations in *NRAS/KRAS* are recurrent in ALL. Besides the well-known hotspot mutations at codons 12, 13, and 61 (i.e., *NRAS* G13D, *KRAS*, and G12D/G13D), novel mutational sites have also been identified in ALL patients by using NGS methods (e.g., *KRAS:* V14I, V14L, K117N and A146T/P). These mutations are usually mutually exclusive, which suggests branching evolution and intra-tumoral heterogeneity [43].

NGS analysis has demonstrated that RAS pathway mutations are common in high hyperdiploid (85%), near-haploid (71%), *KMT2A(MLL1)-*rearrangements (47%), iAMP21 (60%), and ETP ALL (67%) subgroups [24,27,32,33,44,45]. In contrast, RTK- and Ras-signaling alterations are less frequent in low-near-diploid (31.8%), hypodiploid (8.8%), and non-ETP ALL (19%) cases [32]. 

Clinically, some sequencing studies have associated the RAS activating mutations with poor outcomes. Therefore, the presence of these mutations has been recognized as an independent predictor of poor outcome in childhood *KMT2A(MLL1)*-r ALL and the presence of *PTPN11* mutations has been associated with a good prognosis in adult B-ALL patients [46,47]. Not only do RAS mutation-positive patients have a high risk of relapse but also the prognosis for RAS mutation-negative patients remains far from favorable. Therefore, more studies are needed if we are to be able to predict the effect of RAS mutations in ALL patients [47]. The use of MAPK-ERK kinase (MEK) inhibitors and PI3K inhibitors has been proposed as a targeted therapy for the Ras/Raf/MEK/ERK signaling pathway in ALL [23,32].

### 3.3. JAK/STAT Signaling Pathway

Hematopoiesis is the cumulative result of intricately regulated signaling pathways that are mediated by cytokines and their receptors. Through this signaling pathway, cytokines control a variety of important biological responses related to hematopoiesis and immune function including those related to cell growth, differentiation, oncogenesis, and anti-apoptotic signals. The JAK-STAT signaling cascade consists of three main components. These are a cell surface receptor, a Janus kinase (JAK), and a two-signal transducer and activator of transcription (STAT) proteins. Mammals have four Janus kinases Jak1, Jak2, Jak3 (just another kinase) and Tyk2 (tyrosine kinase 2). The binding of various ligands, which are usually cytokines such as interferon (IFNα, IFNγ), interleukin (IL-7, IL-6), and growth factors, to cell surface receptors called GM-CSF activates associated JAKs, which increases their kinase activity. The activated STATs form heterodimers or homodimers and translocate to the cell nucleus where they induce transcription of target genes. The JAK/STAT pathway is negatively regulated at several levels (see Figure 1).

The suppressors of cytokine signaling (SOCS) are a family of STAT target genes that directly antagonize STAT activation, protein tyrosine phosphatases (PTPs) such as SRC homology2, SH2-domain-containing PTP1 (SHP1), and ubiquitin-mediated protein degradation [48]. 

Mutations in genes of this pathway (*JAK1*, *JAK2*, *JAK3*, *IL7R*) have been observed in B-ALL and T-ALL [24,25,49]. Mutations in *JAK* kinases and *IL7R* cause constitutive activation of the JAK-STAT pathway [27]. Activating mutations of JAKs genes (primarily *JAK2* but also *JAK1* and *JAK3*) are also associated with other genetic lesions such as *IKZF1* deletion or mutation and *CRLF2*- rearrangements (*CRFL2*-r) [49,50]. Similarly, loss-of-function mutations in the *SH2B3* gene, which is a negative regulator of *JAK2* signaling, have been identified in ALL. *SH2B3* mutations lead to the loss of regulatory functions and also lead to increased JAK2/STAT signaling [48].

*JAK* mutations are most commonly observed in the pseudokinase domain rather than in the kinase domain. They are usually missense mutations [51]. In particular, the missense mutations at the R683 position are common in the *JAK2* gene [49,51]. The acquired R683 *JAK2* mutations are biomarkers for B-ALL [52,53,54]. Recent studies have suggested that the amino acid residue p.R683 located in the linker between the N and C lobes of the JH2 domain is important for maintaining the activity, structural stability, and folding of JAK2 [55]. The mutations in p.R683 disrupt the structure of the JH2 domain, which leads to the constitutive activation of *JAK2* and the induced growth factor-independent cell proliferation of the mouse Ba/F3 hematopoietic cell line [49,53,55]. At the same time, gain-of-function mutations in the IL7R gene act as oncogenes in T-ALL and B-ALL by generally corresponding to in-frame insertions and indels in the transmembrane domain (e.g., p.L242_L243delinsLCP [49], 243InsPPCL, S185C, 246InsKCH, 241InsFSCGP, 244InsCHL, 244InsPPVCSVT [56], p.Leu242_Leu243insAsnProCys, p.Thr244_Ile245insCysProThr, p.Ile241_Thr244delinsSerAlaAsnCysGlyAla, p.Pro240_Ser246delins LeuGlnSerCys [57]).

*JAK1* mutations have mainly been observed in T-ALL and high-risk B-ALL while *JAK2* mutations have been identified in Down syndrome [52] and high-risk B-ALL [34,54]. In Ph-like ALL, the patients show a high incidence of cytokine-signaling abnormalities. Mutations of *JAK* genes and *CRFL2*-r are often present in Ph-like ALL [50]. ETP ALL has also been characterized by activating mutations in *JAK1*, *JAK3*, *IL7R*, and *SH2B3* [5,34,58].

The constitutively active JAK/STAT signaling pathway results in uncontrolled proliferation of leukemia cells and has been associated with a poor outcome. JAK1/2 inhibitors (e.g., ruxolitinib) and epigenetic drugs could be useful for therapy of patients who harbor mutations in genes involved in the JAK/STAT signaling pathway [54,59]. It has recently been reported that mutations in the IL7R signaling components provide steroid resistance in T-ALL [58]. Therefore, *IL7R* signaling inhibitors could restore or enhance steroid sensitivity in patients with ALL and improve their clinical outcome [58].

### 3.4. TP53 and Cell Cycle Signaling Pathway

The tumor suppressor gene *TP53* is fundamental to cell cycle arrest, apoptosis, DNA repair, and genomic stability and its role in tumorigenesis is well recognized in solid and hematological malignancies. When DNA is damaged, *TP53* is activated and induces cell cycle arrest, which allows the cells to repair the damage. Alternatively, it can induce apoptosis if the DNA damage proves to be irreparable. Under normal conditions, p53 levels are low because it is associated with MDM2, which induces its ubiquitination and destruction by the proteasome [60] (see Figure 1).

*CDKN2A* is an important gene that codes for two proteins, which are ARF and p16. ARF regulates the *TP53* pathway by inhibiting MDM2 and the RB pathway through downregulation of tE2F-1 transcription. In addition, p16 phosphorylates RB, having been phosphorylated disassociates of the transcription factor E2F1, enters the nucleus and promotes the genes essential for transition from the G1 phase to the S phase. Inactivation of *CDKN2A* can lead to deregulation of the *TP53* and *RB* signaling pathways. In fact, both signaling pathways are altered in most human cancer cells [61].

In ALL, genomic alterations of *TP53* (mutation/deletion) are infrequent. Moreover, the *TP53* pathway is also deregulated by abnormalities other than mutations such as hypermethylation of genes involved in the *TP53* cascade, deregulation of microRNAs (e.g., mir-126 and mir-181a), deletion of the *CDNK2A* gene, and overexpression of *MDM2* [62].

In ALL, *TP53* is usually inactivated by missense mutations that are distributed across several exons. The exons are predominantly found in evolutionarily conserved regions of this gene (e.g., L111P, T125R, R110C, H179D, C135R, Y205D, Y220C, M237I, P278S, R273P, C275F, D281N, and R282P) [49]. *TP53* mutations are uncommon in recurrent fusion genes [49,63] and are associated with patients who harbor low hypodiploidy/near triploid and *c-MYC*- rearrangements [5,32,64,65,66]. 

It should be noted that deletions of the *CDKN2A/B* and *RB1* suppressor genes are present in T-ALL (70% and 12%, respectively) and B-ALL (36% and 8%, respectively) in which they contribute to the disruption of the tumor suppressor pathways in ALL [11,67]. Deletions involving *CDKN2A/B* are also common in Ph+ ALL [31]. Genetic alterations involving the *RB1* gene are hallmarks of low-hypodiploid ALL [32].

The clinical importance of *TP53* abnormalities in ALL is tightly linked to poor prognosis and chemorefractoriness [49]. Although the *TP53* mutations are considered infrequent in ALL, these are important when it comes to relapse in childhood and adult ALL and independently predict a high-risk of treatment failure in a significant number of patients [49,68]. The presence of *TP53* mutations is associated with a reduced response rate to induction therapy [63] and with shorter survival [69]. At diagnosis, *TP53* lesions (mutation/deletion) in minor clones may confer resistance to therapy and promote disease relapse [4]. Likewise, recent studies have revealed that *TP53* mutations may also be correlated with a poor prognosis, refractoriness, and unfavorable CR rates in BLs treated with rituximab and intensive chemotherapy [70]. Currently, MDM2-targeted therapy is a promising anticancer alternative for restoring *TP53-*dependent mechanisms in ALL [62].

### 3.5. NOTCH Signaling Pathway

The *NOTCH* pathway is a signaling system in multicellular organisms that regulate cell proliferation, cell fate, differentiation, and apoptosis. The NOTCH receptor genes encode a family of heterodimeric transmembrane proteins (NOTCH1 to NOTCH4) that function as ligand-activated transcription factors. *NOTCH1*, *NOTCH2*, and *NOTCH3* play a pivotal role in committing cell lymphoid precursors to T-cell development. The NOTCH receptor consists of an extracellular subunit, a transmembrane subunit, and an intracellular subunit. Its activation requires cell-to-cell contact. Ligand receptor binding induces cleavage of the transmembrane subunit, which forms an intracellular cleaved form of NOTCH1 by involving the multiprotein protease complex (γ-secretase). Upon activation, the cleaved intracellular portion of the NOTCH receptors translocates into the nucleus and the NIC domain (NICD) binds the DNA-binding protein CSL as well as the SKIP protein. The trimeric complex then recruits the Mastermind-like protein (MAML), which in turn recruits additional co-activators that are required for the transcriptional regulation of the NOTCH target gene expression including the MYC and NF-kβ signaling components. Lastly, the most prominent mechanism of the NOTCH signal suppression operates through its PEST domain of the NICD, which is recognized by the FBXW7 ubiquitin protein ligase and directed towards proteasomal degradation and mediates the termination of NOTCH signaling in the nucleus [28] (see Figure 1).

In T-ALL, the constitutive activation of *NOTCH* signaling is the most prominent oncogenic pathway in T-cell transformation. The activation of this pathway occurs mainly by activating mutations of the *NOTCH1* gene and/or inactivating mutation of the *FBXW7* gene, which confers a strong growth advantage on the leukemic cell [71,72]. Activating mutations in *NOTCH1* have been identified in more than 50% of patients [24,73] and mutations in the *NOTCH2* gene have recently been found especially in adult T-ALL [24]. 

*NOTCH1*-activating mutations localized in the heterodimerization domain (HD) are mostly missense or short in-frame insertions or deletions and result in ligand-independent activation of the receptor while mutations of the negative regulatory PEST domain corresponding to indels increase NICD stability and the consequent half-life of active intracellular NOTCH1, which leads to constitutive activation of the pathway [28,71]. At the same time, *FBXW7*-inactivating mutations are mainly of the missense type and localized at conserved amino-acid positions where they boost NOTCH1 protein stability [24,25].

Oncogenic NOTCH activation is not characteristic of B-ALL, but NOTCH1 and NOTCH2 mutations have been identified in other B-cell malignancies such as chronic lymphocytic leukemia (CLL), splenic marginal zone lymphoma (SMZL), mantle cell lymphoma (MCL), diffuse large B-cell lymphoma (DLBCL) and, rarely, follicular lymphoma (FL) [74].

Although the clinical involvement of *NOTCH1* and *FBXW7* mutations have often been associated with a favorable outcome in T-ALL, its prognostic value is controversial [28]. In T-ALL, *NOTCH1* mutations have been associated with an improved response to glucocorticoids [27]. *NOTCH* inhibitors have been used in combination with inhibitors of the *PI3K/AKT/mTOR* pathway in in vivo assays [27]. Therapeutic antibody targeting of NOTCH1 in T-ALL in combination with other drugs (e.g., glucocorticoids and dexamethasone) as a therapeutic alternative for the clinical management of T-ALL has also been tested with in vitro and in vivo assays [28,75].

### 3.6. PI3K/AKT/mTOR Signaling Pathway

The *PI3K/AKT/mTOR* signaling pathway is mainly involved in regulating cell growth, cell proliferation, cell motility, differentiation, inhibition of apoptosis, and modification of metabolism. In response to the exogenous growth factor or cytokine stimulation, receptor tyrosine kinases (RTKs) or G protein-coupled receptors (GPCRs) are activated and PI3Ks are recruited to the cellular membrane. PI3Ks are divided into three classes on the basis of their structures and substrate specificities [76]. Class IA PI3Ks (*PIK3CA*, *PIK3CB*, and *PIK3CD*) are the most important isoforms in cancer. They consist of a p110 catalytic domain and a non-catalytic domain, p85. PI3K phosphorylates PIP2 generate the second messenger, PIP3. This signaling cascade eventually leads to AKT activation through phosphorylation by PDK1 and the mTORC 1/2 complexes. In turn, AKT phosphorylates and several cellular proteins regulate cellular processes including cell growth. The most important negative control mechanism of this signaling pathway occurs through *PTEN* gene, which mediates the PIP3 transformation to PIP2. This inhibits the downstream cascade [77] (see Figure 1). 

The PI3K/Akt/mTOR signaling pathway is often activated in leukemia and is involved in leukemogenesis [78]. Constitutive activation of this pathway results in enhanced cell metabolism, proliferation, and impaired apoptosis [11]. Its activation is a result of genetic lesions in *PI3K* genes and downstream effectors of the cascade such as *AKT* and *mTOR*. In particular, *PIK3CA* (PI3K-alpha) is a commonly mutated oncogene in T-ALL [11]. Mutational activation and overexpression of this class IA PI3K results in enhanced PI3K signaling, which is associated with oncogenic cellular transformation and cancer [79]. Inactivating mutations in the *PTEN* gene have also been observed in T-ALL patients [24,25]. *PTEN* is the main negative regulator of the PI3K-AKT pathway and its genetic lesions (mutations or deletions) trigger the hyperactivation of this oncogenic pathway in T-ALL due to the increase in AKT1 kinase activity [11].

*PIK3CA* mutations are mainly non-synonymous and arise from single nucleotide substitutions [79]. In addition, missense, silent, and nonsense mutations due to insertions and/or deletions have been described in *PTEN* [11]. 

Alterations of the *PI3K/AKT/mTOR* pathway are predominant in T-ALL (85% of cases) with respect to other leukemia types including B-ALL [80]. Moreover, *PTEN* mutations are common in T-ALL and infrequent in B-ALL [24,81]. In T-ALL patients, sequencing studies of *PTEN* have identified non-synonymous sequence mutations (nonsense or frame-shift mutations) affecting mainly exon 7. Most of these mutations correspond to small insertions or indels that truncate the protein by the premature termination of translation (e.g., Leu247fsX12, Glu242fsX15, Arg234indel, and Gly230fsX12) [82]. 

Within B-ALL, *PI3K/AKT/mTOR* mutations have been identified in near-haploid, low-hypodiploid, and *BCR-ABL* subgroups [83]. A marked induction of mTOR signaling occurs in *CRLF2*-rearrangements in B-ALL [81]. Moreover, although *KMT2A (MLL1)-*r has one of the lowest frequencies of somatic mutations in childhood B-ALL, mutations in kinase-*PI3K-RAS* signaling pathway components (*FLT3*, *KRAS*, *NRAS*, *NF1*, *PTPN11*, *PIK3CA*, and *PIK3R1*) have been described in 47% of these cases [45].

From the clinical point of view, the PI3K/Akt/mTOR signaling pathway has been reported to act in T-ALL, which causes a poor prognosis and a limited response to therapy [78]. *PTEN* mutations have been associated with poor outcome in ALL. *AKT1* mutations promote glucocorticoid resistance, which is an important indicator of therapeutic failure in T-ALL [81]. PI3K inhibitors are currently being developed for clinical use in patients with mutations in genes involved in PI3K-signaling pathways [78,83]. Combined therapy to treat ALL such as *BCR-ABL* TKIs with a panel of selective PI3K/AKT/mTOR inhibitors in *NUP214(CAN)-ABL*-positive T-ALL cell lines [78] and the JAK and PI3K inhibitors ruxolitinib and rapamycin in *CRLF2*-r and JAK-mutated disease [23] are being investigated to maximize the anti-neoplastic effect of different molecular lesions in leukemic cells.

### 3.7. Wnt/β-Catenin Signaling Pathway 

The Wnt signaling pathway is an evolutionarily conserved mechanism that is crucial for the normal development of hematopoietic stem cells (HSCs) and B-cells. Hematopoietic progenitor cells express Wnt proteins and their receptors while responding to Wnt proteins by increasing proliferation. Canonical activation usually involves Wnt protein binding to two receptor molecules, Frizzled (Fzd), which belongs to a class of seven-pass transmembrane receptors and lipoprotein receptor-related proteins 5 or 6 (LRP5/6). Wnt induces the formation of the Fzd-LRP5/6 complex to activate the Wnt signaling pathway. Without Wnt activation, β-catenin (CTNNB1) is phosphorylated by the multi-protein destruction complex and is subject to proteasomal degradation. The cellular concentration of free β-catenin is low because the APC/GSK-3/Axin complex of the adenomatous polyposis coli (APC), the glycogen synthase kinase 3β (GSK-3β), and the axin protein is responsible for regulating the level of β-catenin via GSK-3β-mediated phosphorylation of specific serin and threonine residues in β-catenin. The union of Wnt to its receptors induces the destruction of the β-catenin complex, which falls apart and leads to an excess of β-catenin accumulating in the cytoplasm. Some β-catenin is then able to enter the nucleus and cooperate with the T-cell factor (TCF) transcription factors to activate gene expression of Wnt target genes such as *LEF-1*, *c-MYC*, and cyclin D1. TCF is often in an inactive state in the nucleus. NOTCH and WNT are major players in T-cell development and self-renewal of hematopoietic stem cells pathways. In particular, they are required to turn early T-lineage into single-positive T-cells [84] (see Figure 1).

Inactivating mutations in the Fat cadherin genes *FAT1* and *FAT3* have been described in B-ALL and T-ALL [24,25,46]. These genes encode members of the FAT protocadherin family, which is a group of transmembrane proteins characterized by the presence of cadherin-type repeats. The mutational inactivation of *FAT1* has been linked to the loss of its tumor suppressor capacity and the activation of the WNT pathway [85]. Their inactivating mutations are mainly located within the cadherin domains and are predominantly missense mutations (e.g., R227C, R806C, N1594K, I1719V, and E1769G) [86].

On the other hand, mutations in the *LEF1* gene, which is of great importance in the WNT pathway, have also been described. *LEF1* also presents deletions that are difficult to detect by using NGS methods [24]. *LEF1* mutations are mainly single nucleotide substitutions that generate missense variations (e.g., H86E, K86E, and P106L) [87,88] and have been described in childhood and adult T-ALL [24]. 

Clinically, *LEF1*-inactivating mutations show a favorable trend towards overall survival in pediatric patients with T-ALL, which suggests that this molecular subtype of the disease may be more responsive than other subtypes to salvage therapy for relapsed T-ALL [87].

### 3.8. Chromatin Structure Modifiers and Epigenetic Regulators

Epigenetic mechanisms are essential factors in normal cell development and functioning. Their alteration is a central feature of cancer development. A spectrum of epigenetic regulators and chromatin structure modifiers involved in DNA methylation and histone protein modifications are part of these mechanisms. DNA hyper-methylation leads to the loss of expression of the associated genes. Many important tumor suppressor genes are inactivated by this mechanism. On the other hand, hypo-methylation promotes cancer development through chromosomal instability (by recombination or translocation), reactivation of transposons, and loss of imprinting. Additionally, histone modifications involve acetylation and methylation processes. Acetylation results in a transcriptional activation state while de-acetylation results in a transcriptional loss of expression. The consequences of histone methylation depend on the residue and location modified [89].

ALL may feature somatic mutations in epigenetic regulators such as DNA methylation modifiers (e.g., *DNMT3A*, *TET2*, *IDH1*, and *IDH2*), histone modifiers (e.g., members of polycomb repressive complex 2 [PRC2], such as *SUZ12*, *EZH2*, *EED*, and *EP300*) and histone methyltransferases (e.g., *KMT2D(MLL2)* and *WHSC1*) [11,24]. Likewise, mutations in chromatin structure modifiers such as *PHF6* have been described in ALL [11,90,91]. It should be noted that recurrent inactivating mutations in *SETD2*, *CREBBP*, *KDM6A*, and *NR3C1* have been detected in relapsed ALL [92]. 

Oncogenic activating mutations in epigenetic modifiers (i.e., *IDH1/2*, *EZH2*, and *DNMT3A*) and inactivating mutations in chromatin modifiers (i.e., *KDM6A*, *CREBBP*, *EP300*, and *SMARCB1*) have been observed in ALL [24,93]. Acquired somatic lesions of chromatin structure modifiers and epigenetic regulators correspond to non-synonymous single nucleotide substitutions, frameshift, and nonsense changes [90,91,92,94]. Some of these genes such as *CREBBP*, *EED*, *EZH2*, *KDM6A*, *PHF6*, and *SUZ12* harbor deletions in ALL cases [11,27]. 

Epigenetic alterations are highly prevalent in both B-ALL and T-ALL [5,11,24,34,38,95]. Specifically, B-ALL may feature mutations in histone acetyltransferase *CREBBP* (18% in relapse ALL, rare in cases without high hyperdiploidy), methyltransferases known as *WHSC1* (14–20% in *ETV6-RUNX1* and 15% in rearranged *TCF3-PBX1*), *SETD2* (12%, *KMT2A(MLL1)-* and *ETV6-RUNX1* rearranged), *EZH2* (1.3%, hypodiploidy), phosphorylases known as *JAK2* (10% in high-risk disease, *BCR-ABL*-like, Down syndrome, high-risk disease), *KMT2A(MLL1)* (5%), and *EP300* (<1%) [33,96,97,98]. Somatic mutations of *CREBBP*, *WHSC1*, *KMT5B (SUV420H1)*, *SETD2*, and *EZH2* genes have been identified in high hyper-diploid childhood B-ALL [99,100].

Somatic mutations of genes involved in epigenetic functions and chromatin remodeling such as *PHF6* (11%), *KMT2D(MLL2)* (12%), *DNMT3A* (5%), *TET2* (5%), *SUZ12* (5%), and *EP300* (5%) have been identified in T-ALL [5,11,24,34]. Particularly, somatic mutations in the chromatin structure and epigenetic regulators such as *EZH2*, *SUZ12*, *EED*, *SETD2*, *DNMT3A*, and *EP300* have been found in ETP-ALL [5,24,34]. 

Epigenetic alterations are highly prevalent in ALL and raise the possibility that novel biomarkers could predict patient outcome [95]. *PHF6* and *DNMT3A* mutations are associated with poor outcomes in ALL [35,37]. Alterations (mutation/deletion) in the *CREBBP* gene, which is an important chromatin structure modifier, are acquired at relapse or are already present at diagnosis. They are sometimes located in subclones, which suggests their resistance to therapy (e.g., with glucocorticoids). They also promote disease relapse [4,96]. It has recently been proposed that inhibitors of the epigenetic regulator gene *EZH2* could be useful for the therapy of patients with mutations in that gene [24].

Chromatin modifiers recurrently altered in B-ALL and associated with disease outcomes include *KMT2A(MLL1)*, *CREBBP*, *WHSC1*, and *SETD2.* Clinical trials of drugs potentially targeting histone in ALL patients have been completed and others are underway. For example, in the relapsed and refractory disease, histone deacetylase inhibitors (HDACis) such as FR901228, vorinostat, panobinostat, and *JAK1/JAK2* inhibitors like ruxolitinib have been tested in phase I and II clinical trials [96].

## 4. Landscape of New Structural DNA Rearrangements Detected by NGS

As already indicated, B-ALL and T-ALL comprise heterogeneous subtypes defined by the presence of primary chromosomal abnormalities that are usually chromosomal translocations that help define the particular subtypes of ALL. Although new subtypes of ALL have been recognized in the 2016 revision in the WHO classification of myeloid neoplasms and acute leukemia [10], the classical structural DNA rearrangements described in this disease maintain their biological and clinical importance to their diagnosis and classification and maintain the establishment of therapeutic regimens. 

ALL is the neoplasia in which the greatest number of translocations (more than one thousand) has been described of which more than 150 are recurrent and involve at least 82 fusion genes. Within B-ALL, the structural DNA rearrangements are part of the recurrent genetic abnormalities associated with this disease including hyperdiploidy, hypodiploidy, t(12;21)(p13;q22) *TEL-AML1*(*ETV6-RUNX1*), t(v;11q23) *KMT2A(MLL1)* rearranged, t(1;19)(q23;p13.3) *E2A*(*TCF3)-PBX1*, t(9;22)(q34;q11.2) *BCR-ABL(ABL1)*, t(5;14)(q31;q32) *IL3-IGH*, iAMP21, and *BCR*-*ABL*-like [9]. It is believed that some of the disruptions appear during fetal development especially in the case of childhood leukemia with rearrangements of the *KMT2A (MLL1)* gene and ALL with *TEL-AML1*(*ETV6-RUNX1*) translocation [4]. Important cytogenetic abnormalities in B-ALL that are associated with a poor prognosis include *KMT2A(MLL1)*-*AF4(AFF1)* fusion gene and hypodiploidy with fewer than 45 chromosomes. *BCR-ABL* and Philadelphia chromosome-positive (Ph+) ALL, which is commonly associated with poor outcomes, have improved their prognosis by using TKIs. In contrast, *TEL-AML1(ETV6-RUNX1)* translocation and hyperdiploidy are commonly present in childhood ALL and are subtypes associated with a favorable outcome.

In addition, in T-ALL, the T-cell receptor (TCR) and non-TCR rearrangements involve mostly chromosomal translocations as primary genetic changes in this disease. Unlike B-ALL, the clinical relevance of most subtypes of T-ALL are either unclear or controversial [3]. Gene expression profiling, cytogenetics, and immunophenotypic analyses have been used to identify nonrandom genetic lesions in T-ALL corresponding to rearrangements involving TCR genes (*TCRA/TRAC* (14q11), *TCRB/TRB* (7q34-35), *TCRG/TRG* (7p15), and *TCRD/TRD* (14q11)) and lesions with known oncogenes [101]. These lesions are usually reciprocal translocations and occur at a lower frequency than deletions, duplications, and inversions. Numerical changes are rare except for tetraploidy, which is seen in approximately 5% of cases [102].

TCR genetic lesions lead to the irregular transcription of the partner gene by juxtaposition with the regulatory region of one of the TCR loci [101]. Partner genes usually correspond to important transcription factors involved in T-cell differentiation so their deregulation disrupts normal hematopoiesis and triggers T-ALL [7]. The partner genes usually affected by these rearrangements are *TLX1/HOX11* (10q24), *TLX3/HOX11L2* (5q35), *TAL1/SCL* (1p32), *TAL2* (9q34), *LMO2/RBTN2* (11p13), *LYL1* (19p13), *BHLHB1/OLIG2* (21q22), *LMO1/RBTN1* (11p15), *LCK* (1p34), *NOTCH1/TAN1* (9q34), *c-MYC* (8q24), *TCL1*(14q32), CCND2 (12p13.32), and *MTCP1* (Xq28) [101,102,103,104].

The lesions involving known oncogenes comprise non-TCR rearrangements that result in the formation of ‘fusion genes’ or deletions that are associated with specific T-ALL subgroups [101]. In these translocations, parts of both genes located at the chromosomal breakpoints are fused ‘in frame’ and encode a new chimeric protein with oncogenic properties [104]. The ‘fusion genes’ that mostly define the major subtypes of T-ALL are *SIL(STIL)-TAL1(SCL)*, *PICALM(CALM)-MLLT10(AF10)*, *E2A(TCF3)/PBX1*, *NOTCH1*(*TAN1*)-fusions, *KMT2A(MLL1)*-fusions (*KMT2A(MLL1)*-*MLLT1(ENL)*), *NUP98*-fusions (*NUP98-SETBP1*, *NUP98-RAP1GDS1*, and *NUP98-ADD3*), *ABL1*-fusions (*EML1-ABL1*, *BCR-ABL1*), *NUP214(CAN)-*fusions (*NUP214-ABL1(ABL)*, *SET/NUP214*), and *ETV6*-fusions (*ETV6*(*TEL*)*-JAK2*, *ETV6*(*TEL*)*-ABL1*(*ABL*)) [1,101].

**Figure 3:**
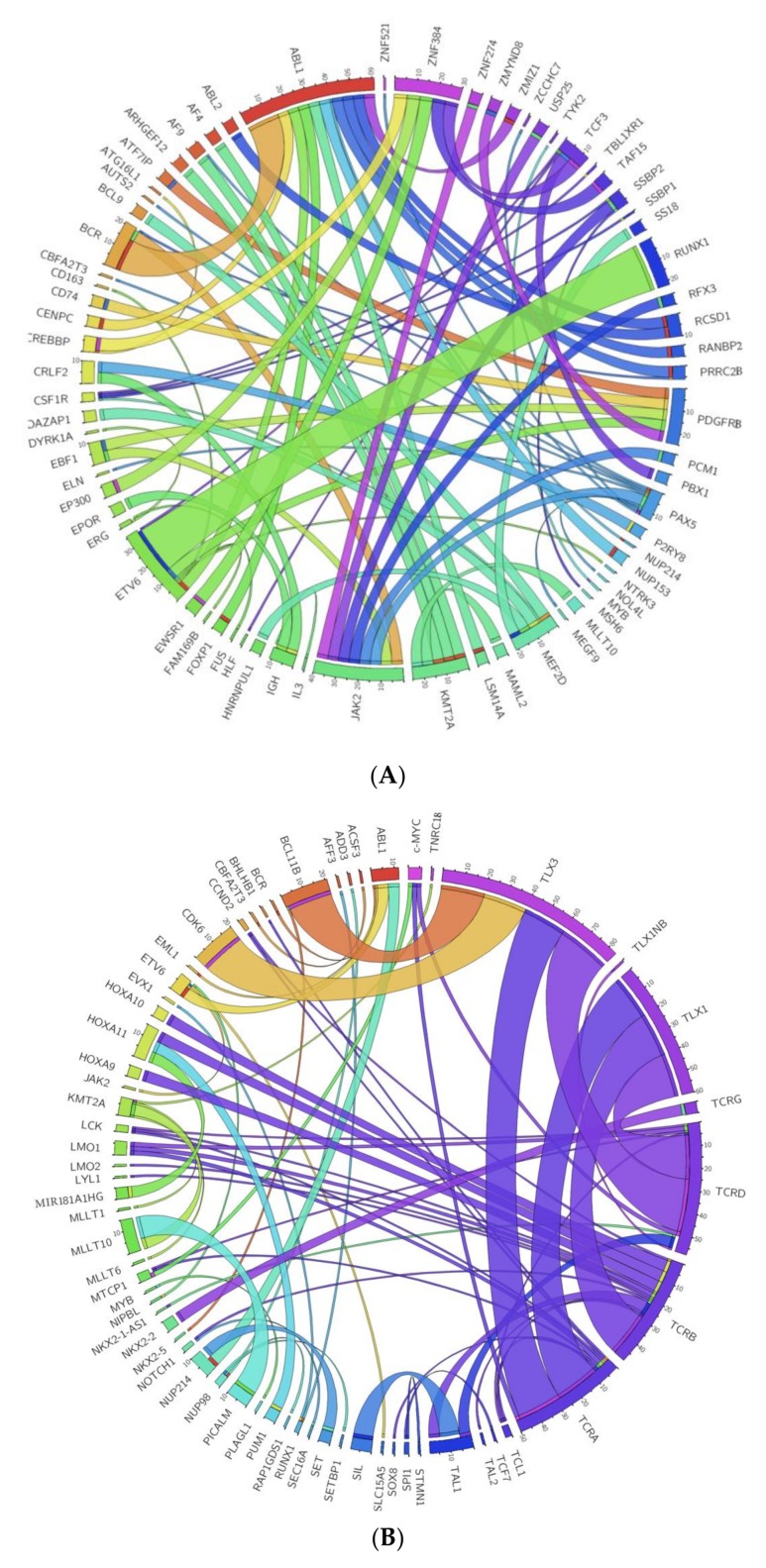
(**A**) New fusion genes identified by NGS approaches in B-ALL. Plot of new fusion genes identified by NGS approaches and their frequencies in B-cell acute lymphoblastic leukemia. (**B**) New fusion genes identified by NGS approaches in T-ALL. Plot of new fusion genes identified by NGS approaches and their frequencies in T-cell acute lymphoblastic leukemia.

Recently, using a range of molecular techniques and NGS approaches such as gene expression, genome-wide sequencing, RNA Kinome Capture, and mRNA-sequencing, a large number of new cryptic DNA rearrangements have been identified in different cohorts of patients with ALL [25,105,106,107,108]. Some of these hidden lesions correspond to novel fusion patterns not identified by cytogenetics or FISH. These fusions involve mostly inter-chromosomal translocations that are caused by new genomic breakpoints and that give rise to distinct fusion transcripts [107]. However, some fusion patterns could also have originated from other types of genetic alterations such as interstitial deletions and inversions [107]. Identifying these new fusion genes provides important information for determining the most appropriate therapeutic strategy for the patient including targeted cancer therapies [107]. Figure 3 shows the fusion genes identified by NGS. The supplemental file 3 details the references of Figure 3. Besides commonly observed fusions, transcriptome sequencing studies have identified novel fusion genes in these B-ALL and T-ALL cohorts. 

Thus, for example, novel recurrent *MEF2D*-fusions (*BCL9*, *HNRNPUL1*, *DAZAP1*) and *ZNF384* fusions (*EP300*, *EWSR1*, *TCF3*, *TAF15*, *CREBBP*) have been described in 6.7% and 7.3% of adults, 3.4% and 3.9% of pediatric patients with B-ALL, respectively [1,105]. In particular, the *MEF2D-BCL9* fusion has recently been found to be associated with a poor prognosis. The leukemic cells with *MEF2D-BCL9* are sensitive to vorinostat and bortezomib in vitro. Therefore, these drugs could be a therapeutic option for these patients [109]. 

Targetable *ABL*-fusions (14.1%; *ABL1(ABL)*, *ABL2*, *CSF1R* and *PDGFRB*), and *EPOR* rearrangements or *JAK2* fusions (8.8%) have been identified in Ph-like ALL patients by RNA-sequencing [15,107]. Specifically, the novel oncogenic fusions identified in Ph-like ALL patients include *CENPC-ABL1*, *LSM14A-ABL1*, *NUP153-ABL1*, *TBL1XR1-CSF1R*, *ZMYND8-PDGFRB*, *GATAD2A-LYN*, *RFX3-JAK2*, *USP25-JAK2*, and *ZNF274-JAK2* [107]. *ABL*-fusions are sensitive to ABL1 TKIs (imatinib and dasatinib) [2] whereas *JAK2*-fusions and *EPOR*-rearrangements are sensitive to ruxolitinib in vitro [107]. It is worth mentioning that *ABL/JAK* class tyrosine kinase activating fusion genes have been found in Ph-like cases [110]. The tyrosine kinase fusions that predict activated ABL signaling include *EBF1-PDGFRB*, *SSBP1-CSF1R*, *ZMIZ1-ABL1*, *FOXP1-ABL1*, and *RCSD1-ABL2* while the tyrosine kinase fusions that predict activated JAK signaling include *PAX5-JAK2*, *BCR-JAK2*, and *TERF2-JAK2* [110].

**Figure 4:**
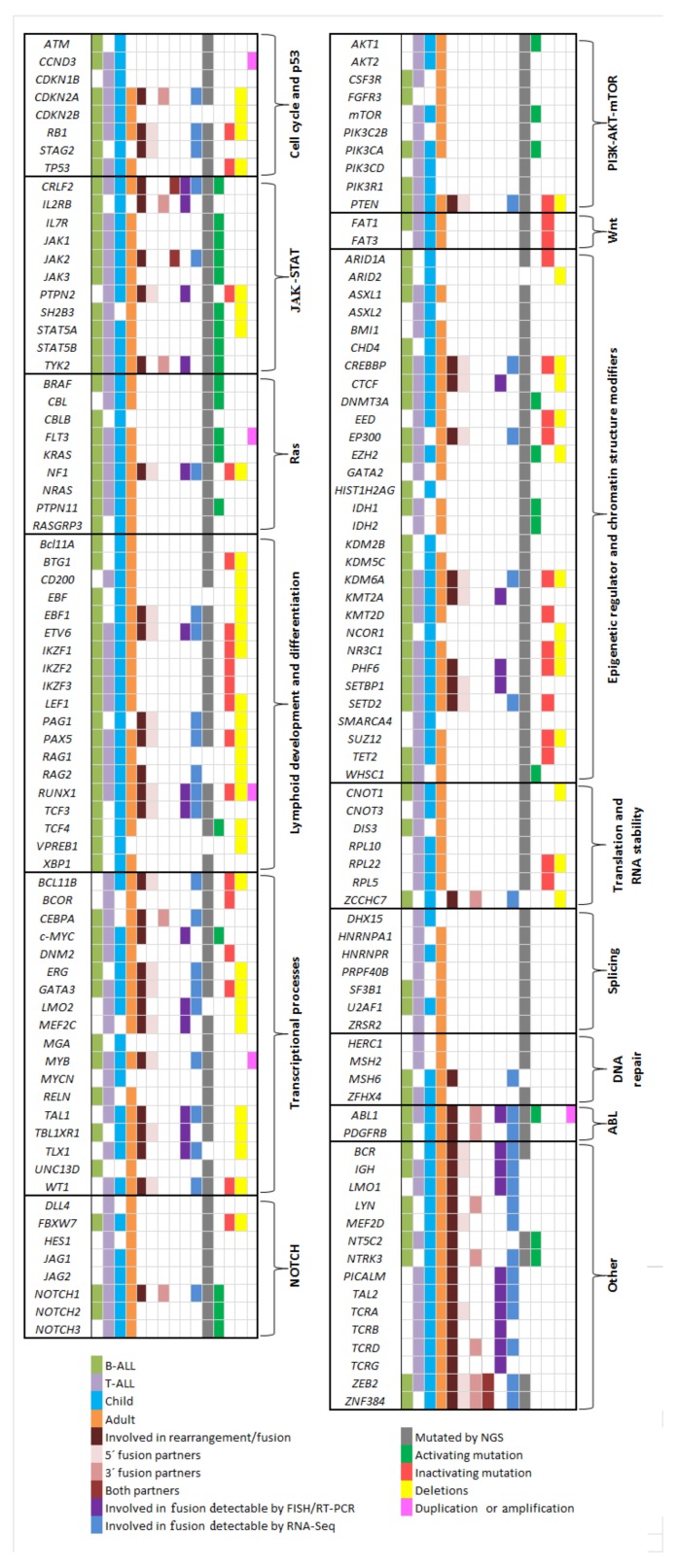
Summary of key genes altered in the different signaling pathways involved in ALL. The figure illustrates the genetic alterations (somatic mutations, rearrangement/fusion, deletions, and duplications) present in the main genes involved in the pathogenic pathways of ALL.

Finally, RNA sequencing analysis has identified gene fusions associated with refractory/relapsed childhood and adult T-ALL. Besides commonly observed fusions (e.g., *TCRB/TRB-LMO2*, *STIL/TAL1(SCL)*, *TCRB/TRB-HOXA10*, *SET-NUP214(CAN)*), RNA-sequencing has revealed new non-recurrent gene fusions (e.g., *HOXA11-AS-MIR181A1HG*, *MAST3-C19orf10/MYDGF*) and rearrangements (e.g., *TCRA*/*TRAC-SOX8*) in T-ALL patients, which indicates that these lesions could be a hallmark of a very poor prognosis in T-ALL [106]. Figure 4 summarizes the key genes altered in the signaling pathways involved in ALL. The supplemental file 4 details the references of Figure 4.

## 5. Conclusions

We have described the application of new NGS technologies for identifying novel cooperative abnormalities in ALL. Some of these lesions could be key prognostic and/or predictive biomarkers for selecting the best frontline treatment and for developing therapy after the first relapse or refractory disease. The identification and validation of new genetic biomarkers may add to or replace the current repertoire of biomarkers available for the clinical management of this disease. 

## Supplementary Materials

The following are available online at http://www.mdpi.com/2072-6694/10/4/110/s1, Supplemental File 1: Table S1. Frequency and prognosis value of cytogenetic and molecular genetic abnormalities identified in ALL; Supplemental File 2: References of Figure 2; Supplemental File 3: References of Figure 3; Supplemental File 4: References of Figure 4.
