# Supplementary material for: New Challenges in Targeting Signaling Pathways in Acute Lymphoblastic Leukemia by NGS Approaches: An Update
Source: Cancers (Basel). 2018 Apr 7;10(4):110. doi: 10.3390/cancers10040110 (PMC5923365; doi:10.3390/cancers10040110)
Supplement: Supplementary file 1 [file cancers-10-00110-s001.zip › cancers-283241-supplement final-/Supplemental file 1.pdf]

Supplemental File 1. Table S1. Frequency and prognosis value of cytogenetic and molecular genetic abnormalities identified in ALL.

Genetic abnormalities [1–8]	Whole cohort [1,9,10]	Pediatric	Adult	Prognosis [3,11–15]
**B-ALL**				
Hyperdiploidy (>50 and <60 chromosomes)	20%-30%	25%	5-10%	Favorable
Near-haploid 24-31 chromosomes	1–3%			Adverse
Low hypodiploid 32-39 chromosomes	1–3%			Adverse
High hypodiploid 40-43 chromosomes	1–3%			Adverse
Near-diploid 44-45 chromosomes				Favorable
TEL-AML1 (ETV6-RUNX1) t(12;21)	15–25%	20–30%	2%	Favorable
KMT2A (MLL) rearranged t(v;11q23)	5–6%	8%	10%	Adverse
E2A-PBX1(TCF3-PBX1) t(1;19)	2–6%	5%	3%	Favorable
BCR-ABL t(9;22)	2–5%	2–4%	25%	Improved *
IL3-IGH@ t(5;14)(q31;q32)	rare	<1%	<1%	Intermediate
CRLF2 rearrangement (IGH-CRLF2; P2RY8-CRLF2)	5–7%			Adverse **
BCR-ABL1-like' or Ph-like	10–15%	10%	20%	Adverse
iAMP21	2%			Adverse
TCF3-HLF t(17;19)	<0.5%			Adverse
DUX4- and ERG-deregulated				Favorable
MEF2D-rearranged		3% to 4%	6%	Unknown
ZNF384-rearranged		3%	7%	Unknown
PAX5 rearrangement	2%			Unknown
**T-ALL**				
**TCR rearrangements**				
TLX1 (HOX11) (10q24)	5–10%	7%	30%	Favorable
TLX3 (HOX11L2) (5q35)	20–25%	20%	10-15%	Adverse ***
TAL1(SCL) (1p32)	15–18%	7%	12%	Favorable
TAL2 (9q34)	<1%			Unknown
LMO1/ RBTN1 (11p15)	10%			Favorable
LMO2/RBTN2 (11p13)	10%			Favorable
LYL1 (19p13)	<1%	1.5%	3%	Adverse
BHLHB1 (21q22)	<1%			Unknown
LCK (1p34)	<1%			Unknown
NOTCH1/TAN1 (9q34)	<1%			Unknown
MYC (8q24)				Unknown
TCL1(14q32)				Unknown
CCND2 (12p13.32)	<1%			Unknown
MTCP1 (Xq28)				Unknown
**Non-TCR rearrangements**				
SIL-TAL1(SCL)	9–30%			Unknown
PICALM(CALM)-MLLT10(AF10)	5–10%			Unknown
E2A/PBX1				Unknown
NOTCH1(TAN1)-fusions				Unknown
KMT2A (MLL)-MLLT1(ENL)	2–5%	0.2%	0.5%	Favorable
KMT2A (MLL) rearrangements	5%			Adverse
NUP98-SETBP1				Unknown
NUP98-RAP1GDS1				Unknown
NUP98-ADD3				Unknown
BCR-ABL1(Ph+)	<1%			Adverse
EML1-ABL1	<1%			Unknown

NUP214(CAN)-ABL1(ABL)	5–15%			Adverse (some studies), intermediate (other studies)
SET/NUP214				Unknown
ETV6(TEL)-JAK2	<1%			Unknown
Early T-cell precursor				Adverse

Abbreviations: ALL= acute lymphoblastic leukemia. * With TKI therapy. ** In non–Down syndrome–associated ALL. *** In some studies.

References

1. Iacobucci, I.; Mullighan, C.G. Genetic basis of acute lymphoblastic leukemia. *J. Clin. Oncol.* **2017**, *35*, 975–983.
2. Chiaretti, S.; Foa, R. T-cell acute lymphoblastic leukemia. *Haematologica* **2009**, *94*, 160–162.
3. Woo, J.S.; Alberti, M.O.; Tirado, C.A. Childhood b-acute lymphoblastic leukemia: A genetic update. *Exp. Hematol. Oncol.* **2014**, *3*, 16.
4. Zhang, L.; Rossi, M.R.; Fisher, K.E. Section ii: Hematolymphoid malignancies. *Curr. Probl. Cancer* **2014**, *38*, 159–174.
5. Hunger, S.P.; Mullighan, C.G. Redefining all classification: Toward detecting high-risk all and implementing precision medicine. *Blood* **2015**, *125*, 3977–3987.
6. Chen, B.; Jiang, L.; Zhong, M.L.; Li, J.F.; Li, B.S.; Peng, L.J.; Dai, Y.T.; Cui, B.W.; Yan, T.Q.; Zhang, W.N.; et al. Identification of fusion genes and characterization of transcriptome features in t-cell acute lymphoblastic leukemia. *Proc. Natl. Acad. Sci. USA* **2018**, *115*, 373–378.
7. Atak, Z.K.; Gianfelici, V.; Hulselmans, G.; De Keersmaecker, K.; Devasia, A.G.; Geerdens, E.; Mentens, N.; Chiaretti, S.; Durinck, K.; Uyttebroeck, A.; et al. Comprehensive analysis of transcriptome variation uncovers known and novel driver events in t-cell acute lymphoblastic leukemia. *PLoS Genet.* **2013**, *9*, e1003997.
8. Neumann, M.; Vosberg, S.; Schlee, C.; Heesch, S.; Schwartz, S.; Gokbuget, N.; Hoelzer, D.; Graf, A.; Krebs, S.; Bartram, I.; et al. Mutational spectrum of adult t-all. *Oncotarget* **2015**, *6*, 2754–2766.
9. Tasian, S.K.; Loh, M.L.; Hunger, S.P. Childhood acute lymphoblastic leukemia: Integrating genomics into therapy. *Cancer* **2015**, *121*, 3577–3590.
10. Taylor, J.; Xiao, W.; Abdel-Wahab, O. Diagnosis and classification of hematologic malignancies on the basis of genetics. *Blood* **2017**, *130*, 410–423.
11. Pui, C.H.; Jeha, S. New therapeutic strategies for the treatment of acute lymphoblastic leukaemia. *Nat. Rev. Drug Discov.* **2007**, *6*, 149–165.
12. Inaba, H.; Greaves, M.; Mullighan, C.G. Acute lymphoblastic leukaemia. *Lancet* **2013**, *381*, 1943–1955.
13. Gowda, C.; Dovat, S. Genetic targets in pediatric acute lymphoblastic leukemia. *Adv. Exp. Med. Biol.* **2013**, *779*, 327–340.
14. Zuckerman, T.; Rowe, J.M. Pathogenesis and prognostication in acute lymphoblastic leukemia. *F1000prime Rep.* **2014**, *6*, 59.
15. Moorman, A.V. New and emerging prognostic and predictive genetic biomarkers in b-cell precursor acute lymphoblastic leukemia. *Haematologica* **2016**, *101*, 407–416.
