# Supplementary material for: New Challenges in Targeting Signaling Pathways in Acute Lymphoblastic Leukemia by NGS Approaches: An Update
Source: Cancers (Basel). 2018 Apr 7;10(4):110. doi: 10.3390/cancers10040110 (PMC5923365; doi:10.3390/cancers10040110)
Supplement: Supplementary file 1 [file cancers-10-00110-s001.zip › cancers-283241-supplement final-/Supplemental file 2.pdf]

Supplemental File 2. References of Figure 2. (Part a) Frequency of somatic mutations identified by NGS methods in childhood and adult B-ALL; **(Part b)** Frequency of somatic mutations identified by NGS methods in childhood and adult T-ALL.

**Part a**					
**Gene Symbol**	**Main pathway**	**Frequency in children with B-ALL**	**Frequency in adult with B-ALL**	**Frequency in whole cohort with B-ALL**	**References**
ADARB1	Others	1%	0%	0,50%	[1]
ASXL1	Epigenetic regulator	1%	3,40%	2,10%	[1–3]
BTG1	Lymphoid development and differentiation	1%	0,60%	0,80%	[1]
c-MYC	Transcriptional processes	1%	2,80%	1,80%	[1,2]
CD3EAP	Others	1%	0,60%	0,80%	[1]
CLDN22	Others	0,50%	1,10%	0,80%	[1]
CREBBP	chromatin structure modifiers	3,40%	2,20%	2.9-5%	[1–3]
EBF1	Lymphoid development and differentiation	0%	1,10%	0,50%	[1,2]
ERBB4	Others	2%	1,10%	1,60%	[1]
ETV6	Lymphoid development and differentiation	2,50%	2,20%	3%	[1,2,4]
EVI5L	Others	1,50%	0,60%	1%	[1]
EZH2	Epigenetic regulator	0,50%	3,40%	1,80%	[1–3]
FLT3	RAS pathway	7%	7%	7%	[1–4]
GNB1	Others	1,50%	3,40%	2,30%	[1]
GPR137C	Others	0,50%	0,60%	0,50%	[1]
HIST1H2AG	Epigenetic regulator	1%	0	0,50%	[1]
IKZF1	Lymphoid development and differentiation	0,50%	3,90%	2,10%	[1,2]
INSRR	Others	1,50%	1,70%	1,60%	[1]
JAK1	Jak-STAT signaling pathway	1%	1,10%	1%	[1,3,4]
JAK2	Jak-STAT signaling pathway	2%	2,20%	2.1-5%	[1,3–5]
JAK3	Jak-STAT signaling pathway	0%	2,20%	1%	[1,3,4]
KCNV1	Others	0,5%	0,60%	0,5%	[1]
KDM5C	Epigenetic regulator	0,50%	0,60%	0,50%	[1]
KRAS	RAS pathway	7,80%	6,10%	7%	[1–4]
MET	Others	3,40%	1,10%	2,30%	[1]
KMT2D	Epigenetic regulator	4,4%	10,1%	5-7%	[1–3]
NF1	RAS pathway	2,90%	6,10%	4,40%	[1–4]
NRAS	RAS pathway	14,70%	8,9%	12-17%	[1–4]
P4HA2	Others	1,50%	1,10%	1,30%	[1]
PABPC3	Others	0%	1,10%	0,50%	[1]
PAX5	Lymphoid development and differentiation	4,90%	9,50%	7%	[1,2,4]

PDE1A	Others	0,50%	0,60%	0,50%	[1]
PHF6	chromatin structure modifiers	0%	1,10%	0,50%	[1]
PROKR1	Others	1%	1,10%	1%	[1]
PTPN11	RAS pathway	6,90%	2,20%	4.7%-8%	[1–4]
RB1	Cell cycle and p53 signaling pathway	1%	0,60%	0,80%	[1,4]
RIT1	Others	0,50%	1,10%	0,80%	[1]
RUNX1	Lymphoid development and differentiation	2%	5%	3,40%	[1,3]
SETD2	Epigenetic regulator	5,90%	7,30%	6,50%	[1,2]
SLC22A6	Others	1%	0,60%	0,80%	[1]
ST6GAL2	Others	0,50%	0,60%	0,50%	[1]
TET2	Epigenetic regulator	0,50%	0,60%	0.5-8%	[1,3]
TP53	Cell cycle and p53 signaling pathway	1%	1,10%	1-4%	[1,2,4,5]
UBA2	Others	2,90%	0,60%	1,80%	[1]
XBP1	Lymphoid development and differentiation	1%	2,20%	1,60%	[1]

Part b.

**Gene Symbol**	**Main pathway**	**Frequency in children with T-ALL**	**Frequency in adult with T-ALL**	**Frequency in whole cohort with T-ALL**	**References**
AKT1	PI3K-AKT-mTOR signaling pathway	2%	2%	2%	[3,6–9]
ASXL2	Epigenetic regulator	2%			[8,9]
BAZ1A	Others	2%			[8]
BCL11B	Transcriptional processes	10%	9%	9%	[7–11]
BCOR	Transcriptional processes		4%		[9,11]
CBL	RAS pathway		5%	<3%	[3,11]
CCND3	Cell cycle and p53 signaling pathway	6%			[8,9]
CDKN1B	Cell cycle and p53 signaling pathway	2%			[8]
CNOT3	Translation and RNA stability	3%	8%		[7–10]
CREBBP	chromatin structure modifiers	2%	~ 10%		[3,8]
CRLF2	Jak-STAT signaling pathway		~ 7%		[3]
CSF3R	PI3K-AKT-mTOR signaling pathway		<3%		[3]
CTCF	Epigenetic regulator	5%			[8,9]
DDX3X	Others	2%			[8]
DHX15	Splicing	2%			[8]

DIS3	Translation and RNA stability		~ 7%		[3]
DLL4	NOTCH pathway		14%		[11]
DNM2	Transcriptional processes	11-13%	13%-17%		[3,7–9,11]
DNMT3A	Epigenetic regulator	1%	6-14%	5%	[7,11]
EED	Epigenetic regulator	5%	5%		[7,9]
EP300	chromatin structure modifiers		5%	5%	[9,11]
ETV6	Lymphoid development and differentiation	3%	4%		[7–9]
EZH2	Epigenetic regulator	5-12%	12%		[3,7–9,11]
FANCA	Others		<3%	<3%	[3]
FAT1	Wnt signaling	2%	16%		[3,8,11]
FAT3	Wnt signaling		14%		[11]
FBXW7	NOTCH pathway	14-24%	11-14%	11-20%	[3,7–11]
FLT3	RAS pathway	5-6%	4%	3%	[3,7,8,11]
GATA2	chromatin structure modifiers		~ 7%		[3]
GATA3	Transcriptional processes	2-5%	3%	<3%	[3,7–9]
HERC1	DNA repair		9%		[11]
HES1	NOTCH pathway		14%		[11]
HUWE1	Others	2%			[8]
IDH1	Epigenetic regulator		10%		[11]
IDH2	Epigenetic regulator		10%		[11]
IL7R	Jak-STAT signaling pathway	7-10%	12%		[7–9,11]
JAG1	NOTCH pathway		14%		[11]
JAG2	NOTCH pathway		14%		[11]
JAK1	Jak-STAT signaling pathway	3-5%	7%		[3,7–9,11]
JAK3	Jak-STAT signaling pathway	8-10%	12-14%		[3,7–9,11]
KDM6A	Epigenetic regulator	5-6%	7%		[8–10]
KMT2A	Epigenetic regulator	2%			[8,9]
KMT2C	Epigenetic regulator	2%			[8,9]
KMT2D	Epigenetic regulator	2%	12%	12%	[8,9,11]
KRAS	RAS pathway	3-6%	5%	16%	[3,7–9,11]
LEF1	Lymphoid development and differentiation	4%	2%		[8,9]
MED12	Others	3%			[8,9]
MTMR3	Others		4%		[11]
mTOR	PI3K-AKT-mTOR signaling pathway		5%		[7,9]
MYB	Transcriptional processes	5%			[8,9]
MYCN	Transcriptional processes	2%			[8]
NOTCH1	NOTCH pathway	50-74%	53-57%	50-56%	[3,7–11]
NOTCH2	NOTCH pathway		14%		[3,11]
NOTCH3	NOTCH pathway		14%		[11]

NRAS	RAS pathway	8-14%	9%-11%	17%	[3,7–9]
ORAI1	Others	2%			[8]
PHF6	chromatin structure modifiers	16%-19%	14%	20%	[3,7–11]
PIK3C2B	PI3K-AKT-mTOR signaling pathway		15%		[11]
PIK3CA	PI3K-AKT-mTOR signaling pathway	1%	5%		[7]
PIK3CD	PI3K-AKT-mTOR signaling pathway	2%			[8]
PIK3R1	PI3K-AKT-mTOR signaling pathway	6%			[8,9]
PRC2	Others		11%		[11]
PRKCZ	Others		4%		[11]
PTEN	PI3K-AKT-mTOR signaling pathway	14-19%	11%	6-8%	[7–9,11]
PTPN2	Jak-STAT signaling pathway	3%	7%		[7]
RELN	Transcriptional processes		<3%	9%	[3,11]
RPL5	Translation and RNA stability	2%	2%		[7,8,10]
RPL10	Translation and RNA stability	6-8%	1%		[7-10]
RPL22	Translation and RNA stability	4%	0%		[7]
RUNX1	Lymphoid development and differentiation	5-8%	10%	9%	[3,7–9,11]
SETBP1	Epigenetic regulator		~ 7%		[3]
SF3B1	Splicing		7%		[3,11]
SH2B3	Jak-STAT signaling pathway		<3%		[3]
SMARCA4	Epigenetic regulator	3%			[8]
STAT5B	Jak-STAT signaling pathway	4-6%	6%		[7–9,12]
SUZ12	Epigenetic regulator	3%	5%	5%	[7–9,11]
TSPYL2	Others	2%			[8]
U2AF1	Splicing	2%	<3%		[3,8]
USP7	Others	12%			[8,9]
USP9X	Others	3%			[8,9]
WHSC1	Epigenetic regulator		5%		[11]
WT1	Transcriptional processes	9-19%	11-12%	10%	[3,7–11]
XIAP	Others		<3%		[3]
ZRSR2	Splicing		4%		[11]

References

1. Liu, Y.F.; Wang, B.Y.; Zhang, W.N.; Huang, J.Y.; Li, B.S.; Zhang, M.; Jiang, L.; Li, J.F.; Wang, M.J.; Dai, Y.J.; et al. Genomic profiling of adult and pediatric b-cell acute lymphoblastic leukemia. *EBioMedicine* **2016**, *8*, 173–183.
2. Zhang, J.; McCastlain, K.; Yoshihara, H.; Xu, B.; Chang, Y.; Churchman, M.L.; Wu, G.; Li, Y.; Wei, L.; Iacobucci, I.; et al. Deregulation of dux4 and erg in acute lymphoblastic leukemia. *Nat. Genet.* **2016**, *48*, 1481–1489.

3. Feng, J.; Li, Y.; Jia, Y.; Fang, Q.; Gong, X.; Dong, X.; Ru, K.; Li, Q.; Zhao, X.; Liu, K. ; et al. Spectrum of somatic mutations detected by targeted next-generation sequencing and their prognostic significance in adult patients with acute lymphoblastic leukemia. *J. Hematol. Oncol.* **2017**, *10*, 61.
4. Roberts, K.G.; Li, Y.; Payne-Turner, D.; Harvey, R.C.; Yang, Y.L.; Pei, D.; McCastlain, K.; Ding, L.; Lu, C.; Song, G.; et al. Targetable kinase-activating lesions in ph-like acute lymphoblastic leukemia. *N. Engl. J. Med.* **2014**, *371*, 1005–1015.
5. Forero-Castro, M.; Robledo, C.; Benito, R.; Bodega-Mayor, I.; Rapado, I.; Hernandez-Sanchez, M.; Abaigar, M.; Maria Hernandez-Sanchez, J.; Quijada-Alamo, M.; Maria Sanchez-Pina, J.; et al. Mutations in tp53 and jak2 are independent prognostic biomarkers in b-cell precursor acute lymphoblastic leukaemia. *Br. J. Cancer* **2017**.
6. Fransecky, L.; Mochmann, L.H.; Baldus, C.D. Outlook on pi3k/akt/mtor inhibition in acute leukemia. *Mol. Cell. Ther.* **2015**, *3*, 2.
7. Girardi, T.; Vicente, C.; Cools, J.; De Keersmaecker, K. The genetics and molecular biology of t-all. *Blood* **2017**, *129*, 1113–1123.
8. Liu, Y.; Easton, J.; Shao, Y.; Maciaszek, J.; Wang, Z.; Wilkinson, M.R.; McCastlain, K.; Edmonson, M.; Pounds, S.B.; Shi, L.; et al. The genomic landscape of pediatric and young adult t-lineage acute lymphoblastic leukemia. *Nat. Genet.* **2017**, *49*, 1211–1218.
9. Seki, M.; Kimura, S.; Isobe, T.; Yoshida, K.; Ueno, H.; Nakajima-Takagi, Y.; Wang, C.; Lin, L.; Kon, A.; Suzuki, H.; et al. Recurrent spi1 (pu.1) fusions in high-risk pediatric t cell acute lymphoblastic leukemia. *Nat. Genet.* **2017**, *49*, 1274–1281.
10. De Keersmaecker, K.; Atak, Z.K.; Li, N.; Vicente, C.; Patchett, S.; Girardi, T.; Gianfelici, V.; Geerdens, E.; Clappier, E.; Porcu, M.; et al. Exome sequencing identifies mutation in cnot3 and ribosomal genes rpl5 and rpl10 in t-cell acute lymphoblastic leukemia. *Nat. Genet.* **2013**, *45*, 186–190.
11. Neumann, M.; Vosberg, S.; Schlee, C.; Heesch, S.; Schwartz, S.; Gokbuget, N.; Hoelzer, D.; Graf, A.; Krebs, S.; Bartram, I.; et al. Mutational spectrum of adult t-all. *Oncotarget* **2015**, *6*, 2754–2766.
12. Kontro, M.; Kuusanmaki, H.; Eldfors, S.; Burmeister, T.; Andersson, E.I.; Bruserud, O.; Brummendorf, T.H.; Edgren, H.; Gjertsen, B.T.; Itala-Remes, M.; et al. Novel activating stat5b mutations as putative drivers of t-cell acute lymphoblastic leukemia. *Leukemia* **2014**, *28*, 1738–1742.
