# Supplementary material for: New Challenges in Targeting Signaling Pathways in Acute Lymphoblastic Leukemia by NGS Approaches: An Update
Source: Cancers (Basel). 2018 Apr 7;10(4):110. doi: 10.3390/cancers10040110 (PMC5923365; doi:10.3390/cancers10040110)
Supplement: Supplementary file 1 [file cancers-10-00110-s001.zip › cancers-283241-supplement final-/Supplemental file 3.pdf]

Supplemental File 3. References of Figure 3. New fusion genes and their frequencies identified by NGS approaches in childhood and adult B-ALL and T-ALL.

Sense of the reference gene	Reference gene	Frequency	Partners 5'	Partners 3'	B-ALL		T-ALL		References
					Children	Adult	Children	Adult	
5'	*IGH*	>5%	*IGH*	*CRLF2*	X				[1–3]
5'		>5%	*IGH*	*EPOR*	X				[1,3–5]
5'	*KMT2A*	>5%	*KMT2A*	*AFF1(AF4)*	X	X			[6]
5'			*KMT2A′*	*ARHGEF12*		X			[6]
5'			*KMT2A*	*MLLT10*			X	X	[7]
5'			*KMT2A*	*AF9 (MLLT3)*	X				[6]
5'			*KMT2A*	*MAML2*	X	X			[8]
5'			*KMT2A*	*MLLT1*			X		[9]
5'			*KMT2A*	*MLLT6*			X		[9]
5'			*KMT2A*	*TNRC18*			X		[9]
5'	*MEF2D*	>5%	*MEF2D*	*BCL9*	X	X			[6,10,11]
5'			*MEF2D*	*DAZAP1*	X	X			[6,10,11]
5'			*MEF2D*	*HNRNPUL1*	X				[6,10,11]
5'			*MEF2D′*	*SS18*	X				[6]
5'	*ETV6(TEL)*	>5%	*ETV6(TEL)*	*RUNX1*	X				[6]
5'		<5%	*ETV6(TEL)*	*SLC15A5*			X		[12]
5'			*ETV6(TEL)*	*NTRK3*	X				[1]
5'			*ETV6(TEL)*	*FAM169B*	X				[5]
5'			*ETV6(TEL)*	*CD163*	X				[5]
5'	*PAX5*	<5%	*PAX5*	*AUTS2*	X				[6]
5'			*PAX5*	*NOL4L*	X	X			[11]
5'			*PAX5*	*CBFA2T3*	X				[6]
5'			*PAX5*	*AF9 (MLLT3)*		X			[6]
5'			*PAX5*	*MEGF9*	X				[3]
5'			*PAX5*	*ZCCHC7*		X			[6]
5'			*PAX5*	*ELN*	X				[5]
5'			*PAX5*	*ZNF521*	X				[5]
5'			*PAX5*	*C20orf112*	X				[5]
3'	*ABCA13*	<5%	*IKZF1*	*ABCA13*			X	X	[13]
3'	*ABL1*	>5% in B-ALL, ≤5% in T-ALL	*BCR*	*ABL1*	X	X	X	X	[6]
3'			*ETV6(TEL)*	*ABL1*	X		X		[1]
3'			*FOXP1*	*ABL1*	X				[14]
3'			*LSM14A*	*ABL1*	X				[1]
3'			*NUP153*	*ABL1*	X				[1]
3'			*NUP214(CAN)*	*ABL1*	X		X	X	[6,13]
3'			*PRRC2B*	*ABL1*		X			[6]
3'			*RANBP2*	*ABL1*	X				[1]
3'			*RCSD1*	*ABL1*	X				[1,2]
3'			*ZMIZ1*	*ABL1*	X				[1]
3'	*ABL1*	>5%	*CENPC*	*ABL1*	X				[1]
3'	*ABL1*	<5%	*ZBTB16*	*ABL1*			X	X	[15]
3'	*ABL2*	>5%	*RCSD1*	*ABL2*	X				[1–3]
3'			*ZC3HAV1*	*ABL2*	X				[1]
3'	*AC016712.2*	<5%	*FARSB*	*AC016712.2*			X	X	[9,13]
3'	*AC093865.1*	<5%	*IKZF2*	*AC093865.1*			X	X	[13]
3'	*ACSF3*	<5%	*CBFA2T3*	*ACSF3*			X		[9]
3'	*AFF3*	<5%	*RUNX1*	*AFF3*			X		[9]
3'	*AHI1*	<5%	*MYB*	*AHI1*			X	X	[13]
3'	*AK7*	<5%	*CREBRF*	*AK7*			X		[9]
3'	*ALAS2*	<5%	*PAGE2B*	*ALAS2*			X	X	[13]
3'	*ANO10*	<5%	*UQCRC1*	*ANO10*		X			[6]
3'	*ARF1*	<5%	*GUK1*	*ARF1*			X	X	[13]

3'	*ARL17A*	<5%	*KIAA1267*	*ARL17A*			X	X	[13]
3'	*ATG16L1*	<5%	*CREBBP*	*ATG16L1*	X				[5]
3'	*ATP6V0C*	<5%	*TSC2*	*ATP6V0C*		X			[6]
3'	*BCR*	<5%	*PRRC2B*	*BCR*		X			[6]
3'	*BMI1*	<5%	*ZEB1*	*BMI1*			X	X	[13]
3'	*C17ORF50*	<5%	*ING4*	*C17ORF50*		X			[6]
3'	*C19orf10*	<5%	*MAST3*	*C19orf10*				X	[12]
3'	*CCDC12*	<5%	*SETD2*	*CCDC12*	X				[5]
3'	*CDK5RAP3*	<5%	*SMARCC1*	*CDK5RAP3*	X				[6]
3'	*CDK6*	<5%	*HOXA11-AS1*	*CDK6*			X	X	[13]
3'	*CRBP1*	<5%	*FGFRL1*	*CRBP1*		X			[6]
3'	*CRLF2 3′*	>5%	*P2RY8*	*CRLF2*	X				[1,2,5]
3'	*CSF1R*	<5%	*SSBP2*	*CSF1R*	X				[1,14]
3'		<5%	*TBL1XR1*	*CSF1R*	X				[1]
		<5%	*SSBP1*	*CSF1R*	X				[14]
3'	*CTBP1*	<5%	*FGFRL1*	*CTBP1*		X			[6]
3'	*CTDP1*	<5%	*PQLC1*	*CTDP1*	X	X			[6]
3'	*CUL2*	<5%	*KDM4B*	*CUL2*	X				[6]
3'	*CXCR4*	<5%	*MGAT4A*	*CXCR4*			X	X	[13]
3'	*DCAF12*	<5%	*UBAP1*	*DCAF12*		X			[6]
3'	*DDX3X*	<5%	*MLLT10*	*DDX3X*			X	X	[13]
3'	*DGKH*	<5%	*CDK1*	*DGKH*	X				[6]
3'	*DNM3*	<5%	*DCAF8*	*DNM3*	X				[6]
3'	*DYRK1A*	<5%	*ERG*	*DYRK1A*	X				[5]
3'	*ERG*	<5%	*FUS*	*ERG*	X				[6]
3'	*ETV6*	<5%	*TCF3*	*ETV6*	X				[5]
3'		<5%	*NIPBL*	*ETV6*			X		[9]
3'	*EVX1*	<5%	*RUNX1*	*EVX1*			X		[9]
3'	*FAS*	<5%	*PTEN*	*FAS*				X	[12]
3'	*FBRSL1*	<5%	*NOC4L*	*FBRSL1*	X				[6]
3'		<5%	*POLE*	*FBRSL1*	X				[6]
3'	*FER*	<5%	*SSBP2*	*FER*			X	X	[13]
3'	*FGFR1*	<5%	*FGFR1OP*	*FGFR1*			X		[9]
3'	*FYTTD1*	<5%	*MFI2*	*FYTTD1*		X			[6]
3'	*GART*	<5%	*RUNX1*	*GART*	X				[6]
3'	*GHR*	<5%	*HDAC4*	*GHR*			X		[9]
3'	*GPR128*	<5%	*TFG*	*GPR128*	X				[5]
3'	*GPR39*	<5%	*MGAT5*	*GPR39*			X	X	[13]
3'	*GS1*	<5%	*GATA3*	*GS1*				X	[12]
3'	*HLF*	<5%	*TCF3*	*HLF*	X				[6]
3'	*HNRNPC*	<5%	*ZNF219*	*HNRNPC*			X	X	[13]
3'	*HOXA10*	>5%	*TCRB*	*HOXA10*			X	X	[10]
3'	*HOXA11*	>5%	*TCRB*	*HOXA11*			X	X	[12]
3'		>5%	*MIR181A1HG*	*HOXA11*			X		[12]
3'		>5%	*PUM1*	*HOXA11*			X		[9]
3'		<5%	*DPY19L1*	*HOXA11*			X	X	[13]
3'	*HOXA9*	>5%	*TCRB*	*HOXA9*			X	X	[10]
3'	*IFNWP19*	<5%	*CDKN2A*	*IFNWP19*			X	X	[13]
3'	*IGH*	<5%	*IL3*	*IGH*		X			[16]
3'	*JAK2 3′*		*BCR*	*JAK2*	X				[1,14]
3'			*EBF1*	*JAK2*	X				[6]
3'			*PAX5*	*JAK2*		X			[1,6,14]
3'			*SSBP2*	*JAK2*	X				[3]
3'			*SNX29*	*JAK2*		X			[6]
3'		>5%	*STRN3*	*JAK2*	X	X			[4]
3'			*PCM1*	*JAK2*	X				[1]
3'			*RFX3*	*JAK2*	X				[1]
3'			*USP25*	*JAK2*	X				[1]
3'			*TERF2*	*JAK2*	X				[14]
3'			*ZNF274*	*JAK2*	X				[1]
3'	*KIF6*	<5%	*KCNK17*	*KIF6*			X	X	[13]
3'	*KIF7*	<5%	*PRC1*	*KIF7*	X				[6]

3'	*LCK*	<5%	*TRD*	*LCK*			X	X	[17]
3'	*LMO1*	<5%	*TCRB*	*LMO1*			X	X	[17]
3'		<5%	*TCRD*	*LMO1*			X	X	[17]
3'	*LMO2*	>5%	*TCRB*	*LMO2*			X	X	[12]
3'	*LYL1*	<5%	*TCRB*	*LYL1*			X	X	[17]
3'	*LYN*	<5%	*GATAD2A*	*LYN*	X				[1]
3'	*MAD1L1*	<5%	*TTYH3*	*MAD1L1*		X			[6]
3'	*MAFG*	<5%	*ASCC1*	*MAFG*			X	X	[13]
3'	*MEF2C*	<5%	*CLINT1*	*MEF2C*			X	X	[13]
3'	*MEF2D*	>5%	*SS18*	*MEF2D*	X				[6]
3'	*METRNL*	<5%	*PIM3*	*METRNL*	X				[6]
3'		<5%	*CSNK1D*	*METRNL*	X				[6]
3'	*MLLT10*	>5%	*PICALM*	*MLLT10*			X		[9]
3'	*MLLT10*	<5%	*DDX3X*	*MLLT10*			X	X	[13]
3'	*MSH6*	<5%	*SSBP2*	*MSH6*	X				[5]
3'	*MTCP1*	<5%	*TCRA*	*MTCP1*			X	X	[18]
3'		<5%	*TCRD*	*MTCP1*			X	X	[18]
3'	*NCLN*	<5%	*PALM*	*NCLN*		X			[6]
3'	*NKD2*	<5%	*SLC12A7*	*NKD2*		X			[6]
3'	*NKX2-2*	>5%	*TCRG*	*NKX2-2*			X		[19]
3'	*NKX2-5*	<5%	*BCL11B*	*NKX2-5*			X		[9]
3'	*NLRP6*	<5%	*RP11-326C3.2*	*NLRP6*			X	X	[13]
3'	*NOTCH1*	<5%	*SEC16A*	*NOTCH1*			X		[9]
3'	*NPR2*	<5%	*HINT2*	*NPR2*	X				[6]
3'	*NUP214*	>5%	*SET*	*NUP214*				X	[9,19]
3'	*NUP98*	<5%	*PSIP1*	*NUP98*			X	X	[13]
3'	*NUTM1*	<5%	*ACIN1*	*NUTM1*	X				[6]
3'	*PAQR6*	<5%	*SMG5*	*PAQR6*				X	[12]
3'	*PAX5*	≤5%	*EMILIN2*	*PAX5*		X			[6]
3'	*PBX1*	>5%	*TCF3*	*PBX1*	X	X			[6]
3'	*PDE9A*	<5%	*WDR4*	*PDE9A*	X				[6]
3'	*PDGFRA*	<5%	*FIP1L1*	*PDGFRA*	X				[3]
3'	*PDGFRB*	<5%	*ETV6(TEL)*	*PDGFRB*		X			[1,4]
3'			*TNIP1*	*PDGFRB*	X				[1,4]
3'			*ZMYND8*	*PDGFRB*	X				[1,4
3'			*CD74*	*PDGFRB*	X				[6]
3'			*EBF1*	*PDGFRB*	X				[1,2,4,14]
3'			*ATF7IP*	*PDGFRB*	X				[1,4]
3'	*PLAGL1*	<5%	*MYB*	*PLAGL1*			X		[9]
3'	*PNRC1*	<5%	*SYNCRIP*	*PNRC1*	X				[5]
3'	*PPP1R36*	<5%	*UBA2*	*PPP1R36*	X				[6]
3'	*PRKAR1B*	<5%	*PCMTD1*	*PRKAR1B*			X	X	[13]
3'	*PSIP1*	<5%	*NUP98*	*PSIP1*			X	X	[13]
3'	*PSMD11*	<5%	*NF1*	*PSMD11*			X		[9]
3'	*PTPRK*	<5%	*L3MBTL3*	*PTPRK*			X	X	[13]
3'	*PVT1*	<5%	*UGCG*	*PVT1*			X	X	[13]
3'	*RAB38*	<5%	*CTSC*	*RAB38*			X	X	[13]
3'	*RBM38*	<5%	*SS18L1*	*RBM38*	X				[5]
3'	*RNF126*	<5%	*OAZ1*	*RNF126*			X	X	[13]
3'	*RP11-326C3.2*	<5%	*NLRP6*	*RP11-326C3.2*			X	X	[13]
3'	*RPL35P4*	<5%	*CDK6*	*RPL35P4*			X	X	[13]
3'	*SCIN*	<5%	*NDUFA4*	*SCIN*		X			[6]
3'	*SET*	<5%	*FUS*	*SET*			X	X	[13]
3'	*SLC7A5*	<5%	*PIM3*	*SLC7A5*	X				[6]
3'		<5%	*CD81*	*SLC7A5*	X				[6]
3'		<5%	*CBFA2T3*	*SLC7A5*	X				[5]
3'	*SLC7A6*	<5%	*CTCF*	*SLC7A6*			X		[9]
3'	*SMARCA2*	<5%	*ZNF362*	*SMARCA2*		X			[6]
3'	*SMCHD1*	<5%	*USP9X*	*SMCHD1*	X				[5]
3'	*SNX29*	>5%	*JAK2*	*SNX29*		X			[6]
3'	*SOX8*	<5%	*TRAC*	*SOX8*				X	[12]

3'	*SPI1*	<5%	*STMN1*	*SPI1*			X		[9]
3'	*SPI1*	<5%	*TCF7*	*SPI1*			X		[9]
3'	*STAT3*	<5%	*STAT5B*	*STAT3*			X	X	[13]
3'	*STIP1*	<5%	*RTN3*	*STIP1*	X				[6]
3'	*TAL1*	>5%	*STIL*	*TAL1*			X	X	[9,12]
3'	*TAL1*	>5%	*TCRB*	*TAL1*			X	X	[12]
3'	*TCRD*	<5%	*TLX1NB*	*TCRD*			X		[9]
3'		<5%	*NKX2-1-AS1*	*TCRD*			X		[9]
3'	*TGIF1*	<5%	*MBP*	*TGIF1*	X				[6]
3'	*THEM7P*	<5%	*WT1*	*THEM7P*				X	[12]
3'	*TMEM259*	<5%	*PTBP1*	*TMEM259*	X				[6]
3'	*TNFSF18*	<5%	*RFWD2*	*TNFSF18*		X			[6]
3'	*TNK2*	<5%	*MFI2*	*TNK2*		X			[6]
3'	*TPM3*	<5%	*B4GALT3*	*TPM3*			X	X	[13]
3'		<5%	*JAK2*	*TPM3*			X	X	[13]
3'	*TRBC1*	<5%	*RIC3*	*TRBC1*			X	X	[13]
3'	*TRBC2*	<5%	*IL7R*	*TRBC2*			X	X	[13]
3'	*TRBJ2-7*	<5%	*PLAG1*	*TRBJ2-7*			X	X	[13]
3'	*TRDC*	>5%	*TAL1*	*TRDC*				X	[12]
3'		<5%	*SFTA3*	*TRDC*			X	X	[13]
3'		<5%	*TLX1NB*	*TRDC*			X	X	[5]
3'	*TSLP*	<5%	*IQGAP2*	*TSLP*	X				[13]
3'	*TYK2*	<5%	*MYB*	*TYK2*	X				[5]
3'	*UFC1*	<5%	*VANGL2*	*UFC1*			X		[9]
3'	*USP9Y*	<5%	*TTTY15*	*USP9Y*				X	[12]
3'	*WDFY2*	<5%	*RB1*	*WDFY2*		X			[6]
3'	*XIST*	<5%	*KDM6A*	*XIST*	X				[5]
3'	*ZBTB18*	<5%	*HNRNPU*	*ZBTB18*	X				[6]
3'	*ZFP36L1*	<5%	*SFPQ*	*ZFP36L1*			X		[9]
3'	*ZNF219*	<5%	*HNRNPC*	*ZNF219*			X	X	[6]
3'	*ZNF362*	<5%	*SMARCA2*	*ZNF362*	X				[13]
3'	*ZNF384 3'*	>5%	*CREBBP*	*ZNF384*	X				[6,10]
3'			*EP300*	*ZNF384*	X	X			[6]
3'			*EWSR1*	*ZNF384*	X	X			[6]
3'			*TAF15*	*ZNF384*		X			[6]
3'			*TCF3*	*ZNF384*	X				[6]

References

1. Reshmi, S.C.; Harvey, R.C.; Roberts, K.G.; Stonerock, E.; Smith, A.; Jenkins, H.; Chen, I.M.; Valentine, M.; Liu, Y.; Li, Y.; et al. Targetable kinase gene fusions in high-risk b-all: A study from the children's oncology group. *Blood* **2017**, *129*, 3352–3361.
2. Yap, K.L.; Furtado, L.V.; Kiyotani, K.; Curran, E.; Stock, W.; McNeer, J.L.; Kadri, S.; Segal, J.P.; Nakamura, Y.; Le Beau, M.M.; et al. Diagnostic evaluation of rna sequencing for the detection of genetic abnormalities associated with ph-like acute lymphoblastic leukemia (all). *Leuk. Lymphoma* **2017**, *58*, 950–958.
3. He, J.; Abdel-Wahab, O.; Nahas, M.K.; Wang, K.; Rampal, R.K.; Intlekofer, A.M.; Patel, J.; Krivstov, A.; Frampton, G.M.; Young, L.E.; et al. Integrated genomic DNA/rna profiling of hematologic malignancies in the clinical setting. *Blood* **2016**, *127*, 3004–3014.
4. Malinowska, I.; Glodkowska-Mrowka, E. Chapter 5—next generation sequencing in hematological disorders. In *Clinical applications for next-generation sequencing*; Academic Press: Boston, MA, USA, 2016; pp 75–96.
5. Roberts, K.G.; Li, Y.; Payne-Turner, D.; Harvey, R.C.; Yang, Y.L.; Pei, D.; McCastlain, K.; Ding, L.; Lu, C.; Song, G.; et al. Targetable kinase-activating lesions in ph-like acute lymphoblastic leukemia. *N. Engl. J. Med.* **2014**, *371*, 1005–1015.
6. Liu, Y.F.; Wang, B.Y.; Zhang, W.N.; Huang, J.Y.; Li, B.S.; Zhang, M.; Jiang, L.; Li, J.F.; Wang, M.J.; Dai, Y.J.; et al. Genomic profiling of adult and pediatric b-cell acute lymphoblastic leukemia. *EBioMedicine* **2016**, *8*, 173–183.

7. Ghazavi, F.; Lammens, T.; Van Roy, N.; Poppe, B.; Speleman, F.; Benoit, Y.; Van Vlierberghe, P.; De Moerloose, B. Molecular basis and clinical significance of genetic aberrations in b-cell precursor acute lymphoblastic leukemia. *Exp. Hematol.* **2015**, *43*, 640–653.
8. Menu, E.; Beaufils, N.; Usseglio, F.; Balducci, E.; Lafage Pochitaloff, M.; Costello, R.; Gabert, J. First case of b all with kmt2a-maml2 rearrangement: A case report. *BMC Cancer* **2017**, *17*, 363.
9. Seki, M.; Kimura, S.; Isobe, T.; Yoshida, K.; Ueno, H.; Nakajima-Takagi, Y.; Wang, C.; Lin, L.; Kon, A.; Suzuki, H.; et al. Recurrent spi1 (pu.1) fusions in high-risk pediatric t cell acute lymphoblastic leukemia. *Nat. Genet.* **2017**, *49*, 1274–1281.
10. Iacobucci, I.; Mullighan, C.G. Genetic basis of acute lymphoblastic leukemia. *J. Clin. Oncol.* **2017**, *35*, 975–983.
11. Suzuki, K.; Okuno, Y.; Kawashima, N.; Muramatsu, H.; Okuno, T.; Wang, X.; Kataoka, S.; Sekiya, Y.; Hamada, M.; Murakami, N.; et al. Mef2d-bcl9 fusion gene is associated with high-risk acute b-cell precursor lymphoblastic leukemia in adolescents. *J. Clin. Oncol.* **2016**, *34*, 3451–3459.
12. Gianfelici, V.; Chiaretti, S.; Demeyer, S.; Di Giacomo, F.; Messina, M.; La Starza, R.; Peragine, N.; Paoloni, F.; Geerdens, E.; Pierini, V.; et al. Rna sequencing unravels the genetics of refractory/relapsed t-cell acute lymphoblastic leukemia. Prognostic and therapeutic implications. *Haematologica* **2016**, *101*, 941–950.
13. Atak, Z.K.; Gianfelici, V.; Hulselmans, G.; De Keersmaecker, K.; Devasia, A.G.; Geerdens, E.; Mentens, N.; Chiaretti, S.; Durinck, K.; Uyttebroeck, A.; et al. Comprehensive analysis of transcriptome variation uncovers known and novel driver events in t-cell acute lymphoblastic leukemia. *PLoS Genet.* **2013**, *9*, e1003997.
14. Boer, J.M.; Steeghs, E.M.; Marchante, J.R.; Boeree, A.; Beaudoin, J.J.; Beverloo, H.B.; Kuiper, R.P.; Escherich, G.; van der Velden, V.H.; van der Schoot, C.E.; et al. Tyrosine kinase fusion genes in pediatric bcr-abl1-like acute lymphoblastic leukemia. *Oncotarget* **2017**, *8*, 4618–4628.
15. Chen, B.; Jiang, L.; Zhong, M.L.; Li, J.F.; Li, B.S.; Peng, L.J.; Dai, Y.T.; Cui, B.W.; Yan, T.Q.; Zhang, W.N.; et al. Identification of fusion genes and characterization of transcriptome features in t-cell acute lymphoblastic leukemia. *Proc. Natl. Acad. Sci. USA* **2018**, *115*, 373–378.
16. Derrieux, C.; Freynet, N.; Frayfer, J.; Delabesse, E.; Clappier, E.; Defasque, S.; Broutier, H.; Fouillard, L. A case of b-cell precursor acute lymphoblastic leukemia with il3-igh rearrangement revealed by thromboembolism and marked eosinophilia. *Leuk. Lymphoma* **2018**, 1–4.
17. Chiaretti, S.; Foa, R. T-cell acute lymphoblastic leukemia. *Haematologica* **2009**, *94*, 160–162.
18. Stern, M.H.; Soulier, J.; Rosenzwajg, M.; Nakahara, K.; Canki-Klain, N.; Aurias, A.; Sigaux, F.; Kirsch, I.R. Mtcp-1: A novel gene on the human chromosome xq28 translocated to the t cell receptor alpha/delta locus in mature t cell proliferations. *Oncogene* **1993**, *8*, 2475–2483.
19. Girardi, T.; Vicente, C.; Cools, J.; De Keersmaecker, K. The genetics and molecular biology of t-all. *Blood* **2017**, *129*, 1113–1123.
