# Supplementary material for: New Challenges in Targeting Signaling Pathways in Acute Lymphoblastic Leukemia by NGS Approaches: An Update
Source: Cancers (Basel). 2018 Apr 7;10(4):110. doi: 10.3390/cancers10040110 (PMC5923365; doi:10.3390/cancers10040110)
Supplement: Supplementary file 1 [file cancers-10-00110-s001.zip › cancers-283241-supplement final-/Supplemental file 4.pdf]

Supplemental File 4. References of Figure 4. Summary of key genes altered in the different signaling pathways involved in ALL.

Pathway	Gene	References
**Cell cycle and p53**	*ATM*	[1,2]
	CCND3	[1,3,4]
	CDKN1B	[3]
	CDKN2A	[1,2,4–7]
	CDKN2B	[1,2,5–11]
	RB1	[1-8,10–13]
	STAG2	[1,5]
	TP53	[1,2,4,5,7,13–15]
**JAK-STAT**	*CRLF2*	[5-7,15,16]
	IL2RB	[5,17]
	IL7R	[2-5,7,8,18,19]
	JAK1	[1-3,5,7-9,16,18,20–22]
	JAK2	[1,5-7,13,15,16,19]
	JAK3	[1,3-5,7,8,13,16,18,19,22,23]
	PTPN2	[8]
	SH2B3	[5,7,16,18,24,25]
	STAT5A	[4]
	STAT5B	[3,4,8,22]
	TYK2	[5,6,17]
**RAS**	*BRAF*	[4,5,13,18]
	CBL	[16,19,26]
	CBLB	[26]
	FLT3	[1-3,5-8,10,13,16,18,26,27]
	KRAS	[1,3-5,7-10,13,16,18,26]
	NF1	[1,2,4,5,13,16,26,28]
	NRAS	[1-4,7-10,13,16,18,26]
	PTPN11	[1,2,5,10,13,16,18,26,29]
	RASGRP3	[30]
**Lymphoid development and differentiation**	*Bcl11A*	[13]
	BTG1	[7,13]
	CD200	[7]
	EBF	[10]
	EBF1	[2,5,6,13]
	ETV6	[1-8,10,13,19]
	IKZF1	[1,3,4,7,10,13]
	IKZF2	[4,9,31]
	IKZF3	[9,31]
	LEF1	[3,4,8,9]
	PAG1	[2,5,31]
	PAX5	[1,5,6,10,13,32]
	RAG1	[32]
	RAG2	[33]
	RUNX1	[1,3-8,13,14,16,18,19,34,35]
	TCF3	[5,36]
	TCF4	[37]
	VPREB1	[38]
	XBP1	[13]
	BCL11B	[3,4,8]

**Transcriptional processes**	*BCOR*	[4,19]
	CEBPA	[16]
	c-MYC	[2,4,6,13]
	DNM2	[3,4,7,8,16,19,22,39]
	ERG	[2,5-7,40]
	GATA3	[2-4,7,8,16,18,22]
	LMO2	[6]
	MEF2C	[19]
	MGA	[2]
	MYB	[3–7]
	MYCN	[3]
	RELN	[16,18,19]
	TAL1	[4,6,8]
	TBL1XR1	[2,7,14]
	TLX1	[4,6]
	UNC13D	[16]
	WT1	[1,3,4,8,9,16,19,41]
**NOTCH**	*DLL4*	[19]
	FBXW7	[1,3,4,6–8,16,19,22]
	HES1	[19]
	JAG1	[19]
	JAG2	[19]
	NOTCH1	[1,3,4,7,8,10,16,19,22,42–44]
	NOTCH2	[16,19]
	NOTCH3	[19]
**PI3K-AKT-mTOR**	*AKT1*	[3,4,8,16,45]
	AKT2	[3]
	CSF3R	[16]
	FGFR3	[16]
	mTOR	[4,8,19]
	PIK3C2B	[3,19]
	PIK3CA	[1,2,8]
	PIK3CD	[3]
	PIK3R1	[2–4]
	PTEN	[1,3,4,7,8,19,22,46]
**Wnt**	*FAT1*	[3,7,16,19,22]
	FAT3	[7,19]
**Epigenetic regulator and chromatin structure modifiers**	*ARID1A*	[1,26]
	ARID2	[2]
	ASXL1	[1,2,4,13,16]
	ASXL2	[3,4]
	BMI1	[7]
	CHD4	[13]
	CREBBP	[1–4,13,16,44,47–50]
	CTCF	[2-4,22,26]
	DNMT3A	[1,6–8,16,19,51–53]
	EED	[4,7,8,18]
	EP300	[1,4,16,18,19,48,50,54,55]
	EZH2	[1-4,8,13,16,18,19,56]
	GATA2	[16,18]
	HIST1H2AG	[13]

	IDH1	[1,16,19]
	IDH2	[1,16,19]
	KDM2B	[2]
	KDM5C	[13]
	KDM6A	[1–5,8]
	KMT2A	[1,3,4]
	KMT2D	[1–4,7,13,19]
	NCOR1	[2]
	NR3C1	[40,57–59]
	PHF6	[1,3,4,6,8,9,13,16,18,19,22,60,61]
	SETBP1	[16]
	SETD2	[1,3–5,13,22]
	SMARCA4	[1,3]
	SUZ12	[3,4,7,8,18,19]
	TET2	[1,13,16,19]
	WHSC1	[2,19,26]
**Translation and RNA stability**	*CNOT1*	[4]
	CNOT3	[3,4,8,19,22]
	DIS3	[16]
	RPL10	[3,4,8,19,22]
	RPL22	[8]
	RPL5	[3,8,19]
	ZCCHC7	[5]
**Splicing**	*DHX15*	[3]
	HNRNPA1	[18]
	HNRNPR	[18]
	PRPF40B	[19]
	SF3B1	[16,19]
	U2AF1	[1–3,16]
	ZRSR2	[19]
**DNA repair**	*HERC1*	[19]
	MSH2	[19]
	MSH6	[40]
	ZFHX4	[13]
**ABL**	*ABL1*	[1,5,6]
	PDGFRB	[5,6,16]
**Other**	*BCR*	[5]
	IGH	[5]
	LMO1	[6]
	LYN	[40]
	MEF2D	[62]
	NT5C2	[7,34,40]
	NTRK3	[5,6,13,17]
	PICALM	[63]
	TAL2	[6]
	TCRA	[64]
	TCRB	[64]
	TCRD	[64]
	TCRG	[64]
	ZEB2	[2,5,6,13,65]
	ZNF384	[2,5]

References

1. He, J.; Abdel-Wahab, O.; Nahas, M.K.; Wang, K.; Rampal, R.K.; Intlekofer, A.M.; Patel, J.; Krivstov, A.; Frampton, G.M.; Young, L.E.; et al. Integrated genomic DNA/rna profiling of hematologic malignancies in the clinical setting. *Blood* **2016**, *127*, 3004–3014.
2. Zhang, J.; McCastlain, K.; Yoshihara, H.; Xu, B.; Chang, Y.; Churchman, M.L.; Wu, G.; Li, Y.; Wei, L.; Iacobucci, I.; et al. Deregulation of dux4 and erg in acute lymphoblastic leukemia. *Nat. Genet.* **2016**, *48*, 1481–1489.
3. Liu, Y.; Easton, J.; Shao, Y.; Maciaszek, J.; Wang, Z.; Wilkinson, M.R.; McCastlain, K.; Edmonson, M.; Pounds, S.B.; Shi, L.; et al. The genomic landscape of pediatric and young adult t-lineage acute lymphoblastic leukemia. *Nat. Genet.* **2017**, *49*, 1211–1218.
4. Seki, M.; Kimura, S.; Isobe, T.; Yoshida, K.; Ueno, H.; Nakajima-Takagi, Y.; Wang, C.; Lin, L.; Kon, A.; Suzuki, H.; et al. Recurrent spi1 (pu.1) fusions in high-risk pediatric t cell acute lymphoblastic leukemia. *Nat. Genet.* **2017**, *49*, 1274–1281.
5. Roberts, K.G.; Li, Y.; Payne-Turner, D.; Harvey, R.C.; Yang, Y.L.; Pei, D.; McCastlain, K.; Ding, L.; Lu, C.; Song, G.; et al. Targetable kinase-activating lesions in ph-like acute lymphoblastic leukemia. *N. Engl. J. Med.* **2014**, *371*, 1005–1015.
6. Taylor, J.; Xiao, W.; Abdel-Wahab, O. Diagnosis and classification of hematologic malignancies on the basis of genetics. *Blood* **2017**, *130*, 410–423.
7. Malinowska, I.; Glodkowska-Mrowka, E. Chapter 5—next generation sequencing in hematological disorders. In *Clinical Applications for Next-Generation Sequencing*; Academic Press: Boston, MA, USA, 2016; pp 75–96.
8. Girardi, T.; Vicente, C.; Cools, J.; De Keersmaecker, K. The genetics and molecular biology of t-all. *Blood* **2017**, *129*, 1113–1123.
9. Iacobucci, I.; Mullighan, C.G. Genetic basis of acute lymphoblastic leukemia. *J. Clin. Oncol.* **2017**, *35*, 975–983.
10. Mrozek, K.; Harper, D.P.; Aplan, P.D. Cytogenetics and molecular genetics of acute lymphoblastic leukemia. *Hematol. Oncol. Clin. North. Am.* **2009**, *23*, 991–1010.
11. Mullighan, C.G.; Downing, J.R. Global genomic characterization of acute lymphoblastic leukemia. *Semin. Hematol.* **2009**, *46*, 3–15.
12. Fang, Q.; Yuan, T.; Li, Y.; Feng, J.; Gong, X.; Li, Q.; Zhao, X.; Liu, K.; Tang, K.; Tian, Z.; et al. Prognostic significance of copy number alterations detected by multi-link probe amplification of multiple genes in adult acute lymphoblastic leukemia. *Oncol. Lett.* **2018**, *15*, 5359–5367.
13. Liu, Y.F.; Wang, B.Y.; Zhang, W.N.; Huang, J.Y.; Li, B.S.; Zhang, M.; Jiang, L.; Li, J.F.; Wang, M.J.; Dai, Y.J.; et al. Genomic profiling of adult and pediatric b-cell acute lymphoblastic leukemia. *EBioMedicine* **2016**, *8*, 173–183.
14. Tijchon, E.; Havinga, J.; van Leeuwen, F.N.; Scheijen, B. B-lineage transcription factors and cooperating gene lesions required for leukemia development. *Leukemia* **2013**, *27*, 541–552.
15. Forero-Castro, M.; Robledo, C.; Benito, R.; Bodega-Mayor, I.; Rapado, I.; Hernandez-Sanchez, M.; Abaigar, M.; Maria Hernandez-Sanchez, J.; Quijada-Alamo, M.; Maria Sanchez-Pina, J.; et al. Mutations in tp53 and jak2 are independent prognostic biomarkers in b-cell precursor acute lymphoblastic leukaemia. *Br. J. Cancer* **2017**.
16. Feng, J.; Li, Y.; Jia, Y.; Fang, Q.; Gong, X.; Dong, X.; Ru, K.; Li, Q.; Zhao, X.; Liu, K.; et al. Spectrum of somatic mutations detected by targeted next-generation sequencing and their prognostic significance in adult patients with acute lymphoblastic leukemia. *J. Hematol. Oncol.* **2017**, *10*, 61.
17. Yokota, T.; Kanakura, Y. Genetic abnormalities associated with acute lymphoblastic leukemia. *Cancer Sci.* **2016**, *107*, 721–725.
18. Zhang, J.; Ding, L.; Holmfeldt, L.; Wu, G.; Heatley, S.L.; Payne-Turner, D.; Easton, J.; Chen, X.; Wang, J.; Rusch, M.; et al. The genetic basis of early t-cell precursor acute lymphoblastic leukaemia. *Nature* **2012**, *481*, 157–163.

19. Neumann, M.; Vosberg, S.; Schlee, C.; Heesch, S.; Schwartz, S.; Gokbuget, N.; Hoelzer, D.; Graf, A.; Krebs, S.; Bartram, I.; et al. Mutational spectrum of adult t-all. *Oncotarget* **2015**, *6*, 2754–2766.
20. Chiaretti, S.; Foa, R. T-cell acute lymphoblastic leukemia. *Haematologica* **2009**, *94*, 160–162.
21. Vainchenker, W.; Constantinescu, S.N. Jak/stat signaling in hematological malignancies. *Oncogene* **2012**, *32*, 2601–2613.
22. Atak, Z.K.; Gianfelici, V.; Hulselmans, G.; De Keersmaecker, K.; Devasia, A.G.; Geerdens, E.; Mentens, N.; Chiaretti, S.; Durinck, K.; Uyttebroeck, A.; et al. Comprehensive analysis of transcriptome variation uncovers known and novel driver events in t-cell acute lymphoblastic leukemia. *PLoS Genet.* **2013**, *9*, e1003997.
23. Bains, T.; Heinrich, M.C.; Loriaux, M.M.; Beadling, C.; Nelson, D.; Warrick, A.; Neff, T.L.; Tyner, J.W.; Dunlap, J.; Corless, C.L.; et al. Newly described activating jak3 mutations in t-cell acute lymphoblastic leukemia. *Leukemia* **2012**, *26*, 2144–2146.
24. Roberts, K.G.; Morin, R.D.; Zhang, J.; Hirst, M.; Zhao, Y.; Su, X.; Chen, S.C.; Payne-Turner, D.; Churchman, M.L.; Harvey, R.C.; et al. Genetic alterations activating kinase and cytokine receptor signaling in high-risk acute lymphoblastic leukemia. *Cancer Cell.* **2012**, *22*, 153–166.
25. Lindqvist, C.M.; Lundmark, A.; Nordlund, J.; Freyhult, E.; Ekman, D.; Carlsson Almlof, J.; Raine, A.; Overnas, E.; Abrahamsson, J.; Frost, B.M.; et al. Deep targeted sequencing in pediatric acute lymphoblastic leukemia unveils distinct mutational patterns between genetic subtypes and novel relapse-associated genes. *Oncotarget* **2016**, *7*, 64071–64088.
26. Ding, L.W.; Sun, Q.Y.; Tan, K.T.; Chien, W.; Thippeswamy, A.M.; Eng Juh Yeoh, A.; Kawamata, N.; Nagata, Y.; Xiao, J.F.; Loh, X.Y.; et al. Mutational landscape of pediatric acute lymphoblastic leukemia. *Cancer Res.* **2017**, *77*, 390–400.
27. Taketani, T.; Taki, T.; Sugita, K.; Furuichi, Y.; Ishii, E.; Hanada, R.; Tsuchida, M.; Sugita, K.; Ida, K.; Hayashi, Y. Flt3 mutations in the activation loop of tyrosine kinase domain are frequently found in infant all with mll rearrangements and pediatric all with hyperdiploidy. *Blood* **2004**, *103*, 1085–1088.
28. Balgobind, B.V.; Van Vlierberghe, P.; van den Ouweland, A.M.; Beverloo, H.B.; Terlouw-Kromosoeto, J.N.; van Wering, E.R.; Reinhardt, D.; Horstmann, M.; Kaspers, G.J.; Pieters, R.; et al. Leukemia-associated nf1 inactivation in patients with pediatric t-all and aml lacking evidence for neurofibromatosis. *Blood* **2008**, *111*, 4322–4328.
29. Tartaglia, M.; Martinelli, S.; Cazzaniga, G.; Cordeddu, V.; Iavarone, I.; Spinelli, M.; Palmi, C.; Carta, C.; Pession, A.; Arico, M.; et al. Genetic evidence for lineage-related and differentiation stage-related contribution of somatic ptpn11 mutations to leukemogenesis in childhood acute leukemia. *Blood* **2004**, *104*, 307–313.
30. Ryan, S.L.; Matheson, E.; Grossmann, V.; Sinclair, P.; Bashton, M.; Schwab, C.; Towers, W.; Partington, M.; Elliott, A.; Minto, L.; et al. The role of the ras pathway in iamp21-all. *Leukemia* **2016**, *30*, 1824–1831.
31. Holmfeldt, L.; Wei, L.; Diaz-Flores, E.; Walsh, M.; Zhang, J.; Ding, L.; Payne-Turner, D.; Churchman, M.; Andersson, A.; Chen, S.C.; et al. The genomic landscape of hypodiploid acute lymphoblastic leukemia. *Nat. Genet.* **2013**, *45*, 242–252.
32. Mullighan, C.G.; Goorha, S.; Radtke, I.; Miller, C.B.; Coustan-Smith, E.; Dalton, J.D.; Girtman, K.; Mathew, S.; Ma, J.; Pounds, S.B.; et al. Genome-wide analysis of genetic alterations in acute lymphoblastic leukaemia. *Nature* **2007**, *446*, 758–764.
33. Van Vlierberghe, P.; van Grotel, M.; Beverloo, H.B.; Lee, C.; Helgason, T.; Buijs-Gladdines, J.; Passier, M.; van Wering, E.R.; Veerman, A.J.; Kamps, W.A.; et al. The cryptic chromosomal deletion del(11)(p12p13) as a new activation mechanism of lmo2 in pediatric t-cell acute lymphoblastic leukemia. *Blood* **2006**, *108*, 3520–3529.
34. Harrison, C.J. Targeting signaling pathways in acute lymphoblastic leukemia: New insights. *Hematology Am. Soc. Hematol. Educ. Program.* **2013**, *2013*, 118–125.
35. Sanda, T. Runx1 in t-all: Tumor suppressive or oncogenic? *Blood* **2017**, *130*, 1686–1688.

36. Chow, R.; Shabsovich, D.; Schiller, G.; Kallen, M.; Tirado, C.A. A t(17;19)(q22;p13.3) involving tcf3, a t(1;9)(p13;p13), and a 5' igh deletion in a case of adult b-cell acute lymphoblastic leukemia. *J. Assoc. Genet. Technol.* **2016**, *42*, 6–14.
37. Kuiper, R.P.; Waanders, E.; van der Velden, V.H.; van Reijmersdal, S.V.; Venkatachalam, R.; Scheijen, B.; Sonneveld, E.; van Dongen, J.J.; Veerman, A.J.; van Leeuwen, F.N.; et al. Ikzf1 deletions predict relapse in uniformly treated pediatric precursor b-all. *Leukemia* **2010**, *24*, 1258–1264.
38. Mangum, D.S.; Downie, J.; Mason, C.C.; Jahromi, M.S.; Joshi, D.; Rodic, V.; Muschen, M.; Meeker, N.; Trede, N.; Frazer, J.K.; et al. Vpreb1 deletions occur independent of lambda light chain rearrangement in childhood acute lymphoblastic leukemia. *Leukemia* **2014**, *28*, 216–220.
39. Tremblay, C.S.; Brown, F.C.; Collett, M.; Saw, J.; Chiu, S.K.; Sonderegger, S.E.; Lucas, S.E.; Alserihi, R.; Chau, N.; Toribio, M.L.; et al. Loss-of-function mutations of dynamin 2 promote t-all by enhancing il-7 signalling. *Leukemia* **2016**, *30*, 1993–2001.
40. Tasian, S.K.; Loh, M.L.; Hunger, S.P. Childhood acute lymphoblastic leukemia: Integrating genomics into therapy. *Cancer* **2015**, *121*, 3577–3590.
41. Tosello, V.; Mansour, M.R.; Barnes, K.; Paganin, M.; Sulis, M.L.; Jenkinson, S.; Allen, C.G.; Gale, R.E.; Linch, D.C.; Palomero, T.; et al. Wt1 mutations in t-all. *Blood* **2009**, *114*, 1038–1045.
42. Weng, A.P.; Ferrando, A.A.; Lee, W.; Morris, J.P.t.; Silverman, L.B.; Sanchez-Irizarry, C.; Blacklow, S.C.; Look, A.T.; Aster, J.C. Activating mutations of notch1 in human t cell acute lymphoblastic leukemia. *Science* **2004**, *306*, 269–271.
43. Zweidler-McKay, P.A.; He, Y.; Xu, L.; Rodriguez, C.G.; Karnell, F.G.; Carpenter, A.C.; Aster, J.C.; Allman, D.; Pear, W.S. Notch signaling is a potent inducer of growth arrest and apoptosis in a wide range of b-cell malignancies. *Blood* **2005**, *106*, 3898–3906.
44. Iacobucci, I.; Papayannidis, C.; Lonetti, A.; Ferrari, A.; Baccarani, M.; Martinelli, G. Cytogenetic and molecular predictors of outcome in acute lymphocytic leukemia: Recent developments. *Curr. Hematol. Malig. Rep.* **2012**, *7*, 133–143.
45. Fransecky, L.; Mochmann, L.H.; Baldus, C.D. Outlook on pi3k/akt/mtor inhibition in acute leukemia. *Mol. Cell. Ther.* **2015**, *3*, 2.
46. Palomero, T.; Sulis, M.L.; Cortina, M.; Real, P.J.; Barnes, K.; Ciofani, M.; Caparros, E.; Buteau, J.; Brown, K.; Perkins, S.L.; et al. Mutational loss of pten induces resistance to notch1 inhibition in t-cell leukemia. *Nat. Med.* **2007**, *13*, 1203–1210.
47. Ueyama, J.I.; Miki, M.; Okuno, K.; Eto, S.; Shimada, A. Msh2 deletion with crebbp and kras mutations in pediatric high-hyperdiploid acute lymphoblastic leukemia. *Pediatr. Int.* **2017**, *59*, 1103–1105.
48. Mullighan, C.G.; Zhang, J.; Kasper, L.H.; Lerach, S.; Payne-Turner, D.; Phillips, L.A.; Heatley, S.L.; Holmfeldt, L.; Collins-Underwood, J.R.; Ma, J.; et al. Crebbp mutations in relapsed acute lymphoblastic leukaemia. *Nature* **2011**, *471*, 235–239.
49. Inthal, A.; Zeitlhofer, P.; Zeginigg, M.; Morak, M.; Grausenburger, R.; Fronkova, E.; Fahrner, B.; Mann, G.; Haas, O.A.; Panzer-Grumayer, R. Crebbp hat domain mutations prevail in relapse cases of high hyperdiploid childhood acute lymphoblastic leukemia. *Leukemia* **2012**, *26*, 1797–1803.
50. Qian, M.; Zhang, H.; Kham, S.K.; Liu, S.; Jiang, C.; Zhao, X.; Lu, Y.; Goodings, C.; Lin, T.N.; Zhang, R.; et al. Whole-transcriptome sequencing identifies a distinct subtype of acute lymphoblastic leukemia with predominant genomic abnormalities of ep300 and crebbp. *Genome Res.* **2017**, *27*, 185–195.
51. Grossmann, V.; Haferlach, C.; Weissmann, S.; Roller, A.; Schindela, S.; Poetzinger, F.; Stadler, K.; Bellos, F.; Kern, W.; Haferlach, T.; et al. The molecular profile of adult t-cell acute lymphoblastic leukemia: Mutations in runx1 and dnmt3a are associated with poor prognosis in t-all. *Genes Chromosomes Cancer* **2013**, *52*, 410–422.
52. Simon, C.; Chagraoui, J.; Krosl, J.; Gendron, P.; Wilhelm, B.; Lemieux, S.; Boucher, G.; Chagnon, P.; Drouin, S.; Lambert, R.; et al. A key role for ezh2 and associated genes in mouse and human adult t-cell acute leukemia. *Genes Dev.* **2012**, *26*, 651–656.

53. Van Vlierberghe, P.; Ambesi-Impiombato, A.; Perez-Garcia, A.; Haydu, J.E.; Rigo, I.; Hadler, M.; Tosello, V.; Della Gatta, G.; Paietta, E.; Racevskis, J.; et al. Etv6 mutations in early immature human t cell leukemias. *J. Exp. Med.* **2011**, *208*, 2571–2579.
54. Tao, Y.F.; Pang, L.; Du, X.J.; Sun, L.C.; Hu, S.Y.; Lu, J.; Cao, L.; Zhao, W.L.; Feng, X.; Wang, J.; et al. Differential mrna expression levels of human histone-modifying enzymes in normal karyotype b cell pediatric acute lymphoblastic leukemia. *Int. J. Mol. Sci.* **2013**, *14*, 3376–3394.
55. Gayther, S.A.; Batley, S.J.; Linger, L.; Bannister, A.; Thorpe, K.; Chin, S.F.; Daigo, Y.; Russell, P.; Wilson, A.; Sowter, H.M.; et al. Mutations truncating the ep300 acetylase in human cancers. *Nat. Genet.* **2000**, *24*, 300–303.
56. Rinke, J.; Muller, J.P.; Blaess, M.F.; Chase, A.; Meggendorfer, M.; Schafer, V.; Winkelmann, N.; Haferlach, C.; Cross, N.C.P.; Hochhaus, A.; et al. Molecular characterization of ezh2 mutant patients with myelodysplastic/myeloproliferative neoplasms. *Leukemia* **2017**, *31*, 1936–1943.
57. Xiao, H.; Wang, L.M.; Luo, Y.; Lai, X.; Li, C.; Shi, J.; Tan, Y.; Fu, S.; Wang, Y.; Zhu, N.; et al. Mutations in epigenetic regulators are involved in acute lymphoblastic leukemia relapse following allogeneic hematopoietic stem cell transplantation. *Oncotarget* **2016**, *7*, 2696–2708.
58. Irving, J.A. Towards an understanding of the biology and targeted treatment of paediatric relapsed acute lymphoblastic leukaemia. *Br. J. Haematol.* **2016**, *172*, 655–666.
59. Irving, J.A.; Enshaei, A.; Parker, C.A.; Sutton, R.; Kuiper, R.P.; Erhorn, A.; Minto, L.; Venn, N.C.; Law, T.; Yu, J.; et al. Integration of genetic and clinical risk factors improves prognostication in relapsed childhood b-cell precursor acute lymphoblastic leukemia. *Blood* **2016**, *128*, 911–922.
60. Van Vlierberghe, P.; Palomero, T.; Khiabanian, H.; Van der Meulen, J.; Castillo, M.; Van Roy, N.; De Moerloose, B.; Philippe, J.; Gonzalez-Garcia, S.; Toribio, M.L.; et al. Phf6 mutations in t-cell acute lymphoblastic leukemia. *Nat. Genet.* **2010**, *42*, 338–342.
61. Mullighan, C.G. Genome sequencing of lymphoid malignancies. *Blood* **2013**, *122*, 3899–3907.
62. Gu, Z.; Churchman, M.; Roberts, K.; Li, Y.; Liu, Y.; Harvey, R.C.; McCastlain, K.; Reshmi, S.C.; Payne-Turner, D.; Iacobucci, I.; et al. Genomic analyses identify recurrent mef2d fusions in acute lymphoblastic leukaemia. *Nat. Commun.* **2016**, *7*, 13331.
63. Savage, N.M.; Kota, V.; Manaloor, E.J.; Kulharya, A.S.; Pierini, V.; Mecucci, C.; Ustun, C. Acute leukemia with picalm-mllt10 fusion gene: Diagnostic and treatment struggle. *Cancer Genet. Cytogenet.* **2010**, *202*, 129–132.
64. Gianfelici, V.; Chiaretti, S.; Demeyer, S.; Di Giacomo, F.; Messina, M.; La Starza, R.; Peragine, N.; Paoloni, F.; Geerdens, E.; Pierini, V.; et al. Rna sequencing unravels the genetics of refractory/relapsed t-cell acute lymphoblastic leukemia. Prognostic and therapeutic implications. *Haematologica* **2016**, *101*, 941–950.
65. Goossens, S.; Radaelli, E.; Blanchet, O.; Durinck, K.; Van der Meulen, J.; Peirs, S.; Taghon, T.; Tremblay, C.S.; Costa, M.; Farhang Ghahremani, M.; et al. Zeb2 drives immature t-cell lymphoblastic leukaemia development via enhanced tumour-initiating potential and il-7 receptor signalling. *Nat. Commun.* **2015**, *6*, 5794.
